# Retinol saturase promotes tubulointerstitial fibrosis in diabetic kidney disease by inhibiting ChREBP ubiquitination via Smurf2 suppression

**DOI:** 10.3389/fendo.2026.1759785

**Published:** 2026-02-04

**Authors:** Heming Huang, Wei Xu, Yang Wang, Yanjia Shi, Sijing Tang, Ping Fang, Lingling Pan, Zhengqin Ye, Yun Zhou, Jieli Huang, Ying Xue

**Affiliations:** 1 Department of Endocrinology and Metabolism, Tongji Hospital, School of Medicine, Tongji University, Shanghai, China; 2 Department of Geriatric Medicine, Tongji Hospital, School of Medicine, Tongji University, Shanghai, China; 3 Department of Nephrology, Tongji Hospital, School of Medicine, Tongji University, Shanghai, China

**Keywords:** diabetic kidney disease, retinol saturase, Smurf2, tubulointerstitial fibrosis, ubiquitination

## Abstract

**Background:**

Renal tubulointerstitial fibrosis (TIF) is a hallmark pathological feature of diabetic kidney disease (DKD). This study investigates the role and molecular mechanisms of retinol saturase (RetSat) in DKD-associated TIF.

**Methods:**

RetSat expression was assessed in renal tissues from DKD patients and mice and correlated with the severity of TIF. Functional experiments were conducted *in vitro* using HK2 cells to evaluate the effects of RetSat overexpression and knockdown on high-glucose-induced tubular injury and fibrosis. Mechanistically, we examined the expression of the E3 ubiquitin ligase SMAD ubiquitination regulatory factor 2 (Smurf2), carbohydrate-responsive element-binding protein (ChREBP), and various fibrosis markers. Furthermore, the protein-protein interaction and ubiquitination relationship between RetSat and Smurf2 were explored.

**Results:**

RetSat expression was significantly up regulated in the renal tissues of both DKD patients and mice, correlating with the deterioration of TIF. *In vitro*, RetSat overexpression exacerbated high-glucose-induced tubular injury and fibrosis in HK2 cells, whereas RetSat knockdown attenuated these pathological phenotypes. Mechanistically, RetSat interacted with Smurf2 and promoted its degradation via ubiquitination. This reduction in Smurf2 subsequently prevented the Smurf2-mediated ubiquitination of ChREBP, leading to ChREBP accumulation and the up regulation of tubular injury and fibrosis markers.

**Conclusion:**

These findings indicate that RetSat promotes TIF in DKD by disrupting the Smurf2-ChREBP ubiquitination axis, highlighting RetSat as a promising therapeutic target for DKD.

## Introduction

Diabetic kidney disease (DKD) is the leading cause of chronic kidney disease (CKD) and end-stage renal disease (ESRD) globally (1). Approximately 40% of diabetic patients develop DKD (2), making its clinical management a significant challenge (3). Pathologically, DKD is defined by glomerulosclerosis, tubular inflammation, atrophy, and interstitial fibrosis (4). Among these features, tubulointerstitial fibrosis (TIF) is a critical determinant of the progression to ESRD and is characterized by the irreversible loss of renal function (5, 6). Early-stage DKD often manifests as hypertrophy and hyperplasia of renal tubular epithelial cells alongside tubular basement membrane thickening, changes that act as key drivers of TIF initiation (7, 8). Furthermore, persistent hyperglycemia, combined with ischemia and hypoxia, promotes tubular cell apoptosis, atrophy, and degeneration (9). Despite these observations, the precise molecular mechanisms underlying the onset and progression of DKD-associated TIF remain incompletely understood (5, 6).

Ubiquitination is a ubiquitous post-translational modification that regulates protein stability, cellular localization, and biological activity (10). Within this pathway, E3 ubiquitin ligases are pivotal, as they dictate the specific recognition of target essential for cellular homeostasis. Emerging evidence underscores the critical role of ubiquitination in DKD pathogenesis. Specifically, numerous E3 ubiquitin ligases modulate renal epithelial-mesenchymal transition (EMT), inflammation, and fibrosis via specific signaling cascades (11–13). Consequently, E3 ligases and their substrates are increasingly recognized as potential therapeutic targets for DKD (11–13).

Retinol saturase (RetSat) is an evolutionarily conserved oxidoreductase highly expressed in adipose tissue, liver, and kidney (14). Localized primarily in the endoplasmic reticulum, RetSat catalyzes the conversion of retinol to 13,14-dihydroretinol (13,14-dhretinol) (15). Its transcription is regulated in a tissue-specific manner by peroxisome proliferator-activated receptor α (PPARα) in the liver (16), PPARγ in adipose tissue (15), and forkhead box O1 (FOXO1) in hepatocytes (17). RetSat has been implicated in insulin resistance and type 2 diabetes mellitus (T2DM) (18), governing processes such as adipocyte differentiation, hepatic metabolism, and macrophage function (15, 19, 20). Notably, RetSat contributes to fatty liver disease by regulating carbohydrate-responsive element-binding protein (ChREBP) in a manner independent of its enzymatic product, dehydroretinol (21). Despite its established role in metabolic regulation, the specific functions and regulatory mechanisms of RetSat in DKD, particularly regarding TIF, remain largely unexplored.

This study reveals for the first time that RetSat and its associated ubiquitination network play a critical role in the pathogenesis of DKD-related TIF. We show that RetSat promotes TIF by up-regulating ChREBP expression in renal tubules. Mechanistically, RetSat does not directly target ChREBP; rather, it promotes the self-ubiquitination and degradation of the E3 ubiquitin ligase SMAD ubiquitin regulatory factor 2 (Smurf2). The consequent reduction in Smurf2 prevents ChREBP degradation, leading to its accumulation. This study elucidates the novel RetSat-Smurf2-ChREBP regulatory axis, providing new insights into DKD pathogenesis and identifying potential therapeutic targets.

## Materials and methods

### Human samples

This study was approved by the Ethics Committee of Tongji Hospital (No. K-W-2024-009). Renal biopsy samples were obtained from 15 patients, comprising 7 with DKD and 8 with minimal change disease (MCD) as controls. Details were provided in the **Supplementary Materials**.

### Animal study

Eight-week-old male C57BL/6 mice were randomly assigned to either the normal control group (NC, n=6) or DKD (n=6) group. Kidney tissues were harvested for subsequent analysis. All animal procedures were ethically approved and conducted under specific pathogen-free conditions. Detailed procedures were provided in the **Supplementary Materials**.

### Cell culture and treatment

HK2 and HEK293T cells were cultured under standard conditions. To establish an *in vitro* DKD model, HK2 cells were serum-starved for 24 hours and subsequently treated with high glucose (HG, 30 mM) or normal glucose (NG, 5.5 mM) for 72 hours. To investigate the regulatory role of RetSat and Smurf2 on ChREBP, HK2 cells were transfected with specific overexpression vectors. Detailed transfection protocols are provided in the **Supplementary Materials**.

### Small interfering RNA transfection

To investigate the function of RetSat, HK2 cells were transfected with RetSat-targeting siRNA using Lipo8000™ reagent. siRNA sequences and detailed transfection procedures are provided in the **Supplementary Materials**.

### Co-immunoprecipitation

To assess protein-protein interactions, HEK293T cells were transfected with the indicated vectors. Lysates were subjected to co-immunoprecipitation using specific antibody-conjugated beads, followed by Western blot analysis. Detailed procedures were provided in the **Supplementary Materials**.

### Ubiquitination assay

HEK293T cells were co-transfected with Myc-Ub, Flag-RetSat, Ha-Smurf2, and Myc-ChREBP plasmids, followed by treatment with MG132 or Heclin to assess ubiquitination status. Protein interactions and ubiquitination levels were analyzed by Co-IP and SDS-PAGE, as detailed in the **Supplementary Materials**.

### Western blotting analysis

Total protein was extracted from kidney tissues or HK2 cells using RIPA buffer and quantified via BCA assay. Lysates were resolved by SDS-PAGE and immunblotted with specific primary antibodies followed by HRP-conjugated secondary antibodies. Antibody details and protocols are listed in the **Supplementary Materials**.

### Quantitative real-time PCR

Total RNA was extracted from tissues or cells and reverse-transcribed into cDNA. qPCR was performed using SYBR Green with β-actin serving as the internal control. Primer sequences and protocols are available in the **Supplementary Materials** and **Supplementary Table S1**.

### AlphaFold2 prediction

Protein sequences for RetSat, ChREBP, and Smurf2 were retrieved from UniProtKB and PDB. Full-length structures were predicted using AlphaFold via Google Colab. The highest-confidence models were selected and visualized using UCSF ChimeraX (22–24). Futher details are provided in the **Supplementary Materials**.

### Histological analysis

Kidney tissues were paraffin-embedded and stained with H&E, PAS, PASM, and Sirius Red. Morphological changes, including mesangial expansion, tubular injury, and fibrosis, were quantified using ImageJ. Additionally, ultrastructural analysis of human kidney tissues was performed using transmission electron microscopy (TEM) at 8000 × and 30000× magnification. Detailed scoring methods and procedures were provided in the **Supplementary Materials**.

### Immunofluorescence

Immunofluorescence staining was performed as previously described (25). Kidney sections were incubated with anti-RetSat antibody and followed by Alexa Fluor 594-conjugated secondary antibody. Nuclei were stained with DAPI. IgG deposition was detected using an Alexa Fluor 488-conjugated anti-IgG antibody. Images were captured using an inverted fluorescence microscope, as detailed in the **Supplementary Materials**.

### Biochemical measurements

Serum creatinine (Scr) and blood urea nitrogen (BUN) were measured using an automated biochemical analyzer. Urinary kidney injury molecule-1 (KIM-1) levels were quantified via ELISA. Protocols are described in the **Supplementary Materials**.

### Label-freequantification-based proteomics analysis

Renal proteins (n=3 mice/group) were extracted and digested using the filter-aided sample preparation (FASP) method. Peptides were analyzed by LC-MS/MS for label-free quantification. Detailed mass spectrometry parameters are provided in the **Supplementary Materials**.

### GEO dataset analysis

Gene expression data from DKD and control kidney samples (GSE228960) were analyzed using R. Differential expression analysis and Gene Set Enrichment Analysis (GSEA) were performed to identify enriched pathways. Data processing details are in the **Supplementary Materials**.

### Single-nucleus RNA-seq data processing and analysis

Publicly available snRNA-seq datasets (GSE195460, GSE131882, GSE151302) were re-analyzed using the Seurat R package (26) to characterize the cellular landscape, quantify cell type proportions, and evaluate RETSAT expression patterns. Analysis parameters are detailed in the **Supplementary Materials**.

### Immunoprecipitation mass spectrometry analysis

HEK293T cells were transfected with Flag or Flag-RetSat plasmids. Interacting proteins were isolated via Flag-immunoprecipitation, identified by LC-MS/MS, and analyzed using MaxQuant. The interactome was compared with TGF-β signaling pathway genes, and overlaps were visualized using Venn diagrams. Full details were in the **Supplementary Materials**.

### Statistical analysis

Statistical analyses were conducted using SPSS software (Version 27.0; IBM, USA), R software (Version 4.2.1, The R Foundation; http://www.R-project.org), and GraphPad Prism 9.0 (GraphPad Software, Inc., La Jolla, CA, USA). Data were expressed as means ± standard deviations (SD). Differences between two groups were evaluated using the unpaired, two-tailed Student’s t-test, assuming a normal distribution of the data. p-values < 0.05 were considered statistically significant, and all tests were two-sided.

## Results

### RetSat is up-regulated in the kidneys of DKD mice

**Figure 1:**
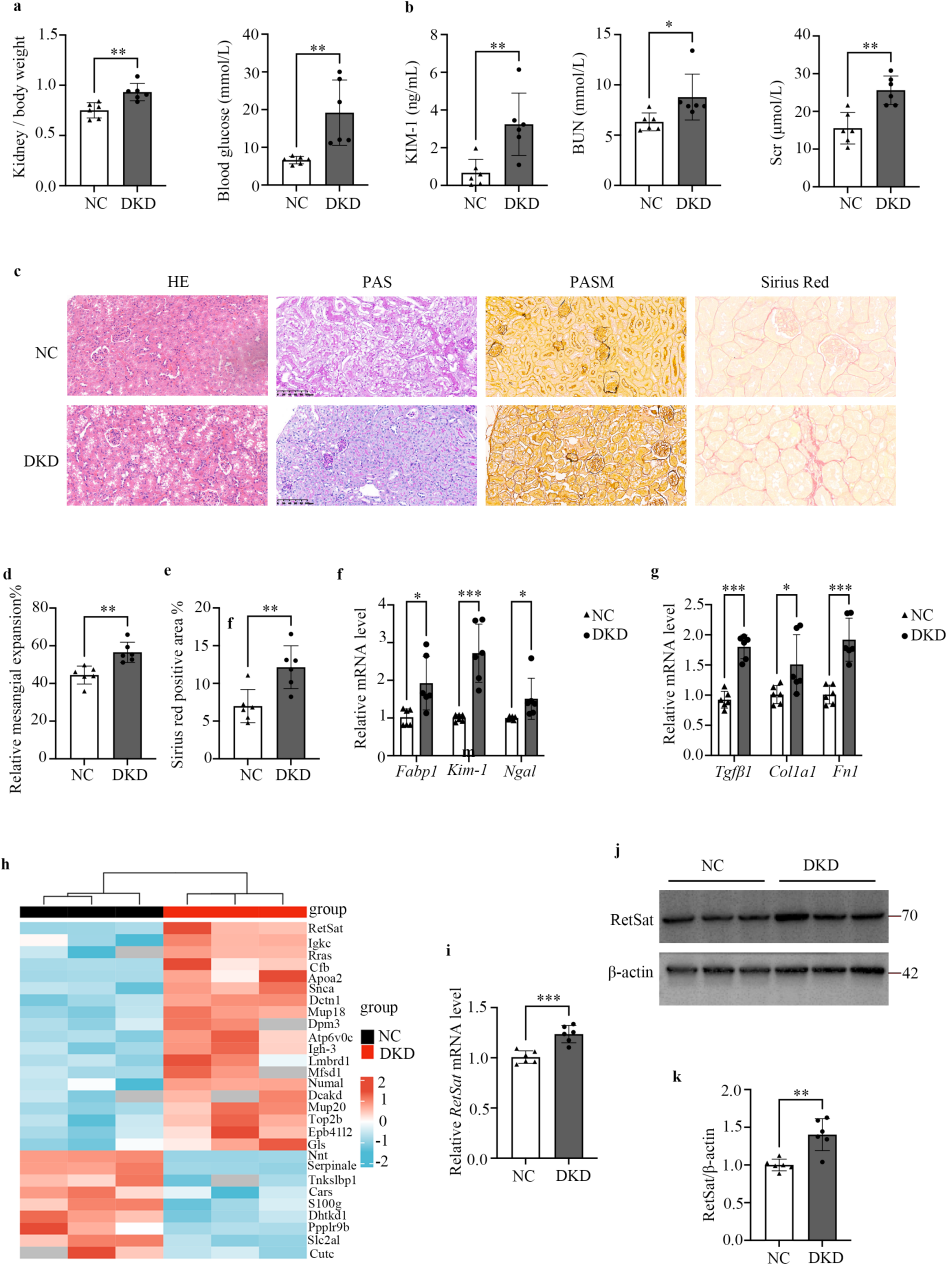
RetSat is up-regulated in the kidneys of DKD mice. **(a)** Kidney weight-to-body weight ratio and blood glucose of mice in NC and DKD groups (n=6). **(b)** Levels of urinary KIM-1, BUN and Scr in the NC and DKD groups (n=6). **(c)** Representative images of HE, PAS, PASM and Sirius Red staining of kidney sections (200× magnification). **(d)** Relative quantitative analysis of mesangial expansion (n=6). **(e)** Quantitative analysis of the Sirius Red-positive area (n=6). **(f)** Relative mRNA expression of the tubular injury markers *Fabp1*, *Kim-1*, and *Ngal* in kidney tissues (n=6). **(g)** Relative mRNA expression of the fibrosis markers *Tgfβ1*, *Col1α1*, and *Fn1* in kidney tissues (n=6). **(h)** Heatmap visualization of differentially expressed proteins between the NC and DKD groups (n=3). **(i)** Relative mRNA expression of RetSat in kidney tissues (n=6). **(j)** Representative Western blot of RetSat protein expression in kidney tissues. **(k)** Semi-quantitative analysis of RetSat protein levels. Data are presented as mean ± SD. *p< 0.05, **p< 0.01, ***p < 0.001 vs. NC group.

A DKD mouse model was established via STZ injection combined with a high-fat diet (HFD). Compared to the NC group, DKD mice exhibited a significantly higher kidney weight-to-body weight ratio and elevated blood glucose levels (**Figure 1a**). Additionally, urinary KIM-1, BUN, and Scr levels were markedly increased in the DKD group (**Figure 1b**). Histological analysis using H&E, PAS, and PASM staining revealed significant glomerular and tubular damage in DKD mice. Specifically, glomerular mesangial expansion, tubular epithelial disorganization, capillary basement membrane thickening, and vacuolar degeneration were more pronounced in the DKD mice than in the NC group (**Figure 1c**, columns 1–3, **1d**). Furthermore, Sirius Red staining demonstrated renal tubular atrophy and interstitial fibrosis in the DKD group (**Figure 1c**, column 4, **1e**). Consistent with these histological findings, qPCR analysis showed that mRNA levels of the renal tubular injury markers fatty acid binding protein 1 (Fabp1), Kim-1, and neutrophil gelatinase-associated lipocalin (Ngal) were significantly elevated in kidney tissues of DKD mice (**Figure 1f**). Similarly, the expression of fibrosis markers collagen type I alpha 1 chain (Col1a1), fibronectin 1 (Fn1), and transforming growth factor beta 1 (Tgfβ1) was significantly up-regulated in DKD kidneys (**Figure 1g**). Collectively, these results confirm the successful establishment of the DKD mouse model.

To explore the underlying pathogenesis and identify potential therapeutic targets, we performed label-free quantitative (LFQ) proteomics analysis to screen for differentially expressed proteins (DEPs) in kidney tissues. Heatmap analysis identified 30 significant DEPs (P < 0.05), comprising 19 up-regulated and 11 down-regulated proteins in the DKD group compared to controls (**Figure 1h**). Notably, RetSat was significantly up-regulated in the DKD group and exhibited the most profound difference in expression between the two groups. Subsequent *in vivo* validation confirmed that both RetSat mRNA (**Figure 1i**) and protein levels (**Figures 1j, k**) were markedly increased in DKD kidneys compared to controls. These findings suggest that RetSat may play a critical role in DKD pathogenesis. To validate this in an independent model, we analyzed the GSE228960 dataset (db/db mice) from the GEO database. Heatmap analysis confirmed that RetSat was significantly up-regulated in the kidneys of the DKD group (**Supplementary Figure S1A**). Furthermore, GSEA of this dataset revealed significant enrichment of the collagen fiber pathway in DKD kidney tissues (**Supplementary Figure S1B**). These results underscore the importance of fibrosis in DKD progression and suggest the potential involvement of RetSat in this process.

### RetSat is highly expressed in renal tubules of DKD patients

Comparative analysis of clinical characteristics revealed no significant differences in age, sex distribution, or body mass index (BMI) between the DKD and MCD groups (**Supplementary Table S2**). Notably, glycated hemoglobin A1c (HbA1c) levels were significantly elevated in the DKD group compared to the MCD group (7.1 ± 1.2% vs. 6.0 ± 0.4%, p=0.026). Although serum creatinine and blood urea nitrogen (BUN) levels were numerically higher in DKD patients, the differences between the two groups were not statistically significant. Similarly, no significant differences were observed between the two groups in estimated glomerular filtration rate (eGFR) and urine albumin-to-creatinine ratio (UACR), indicating comparable degrees of renal functional impairment and proteinuria in both groups.

**Figure 2:**
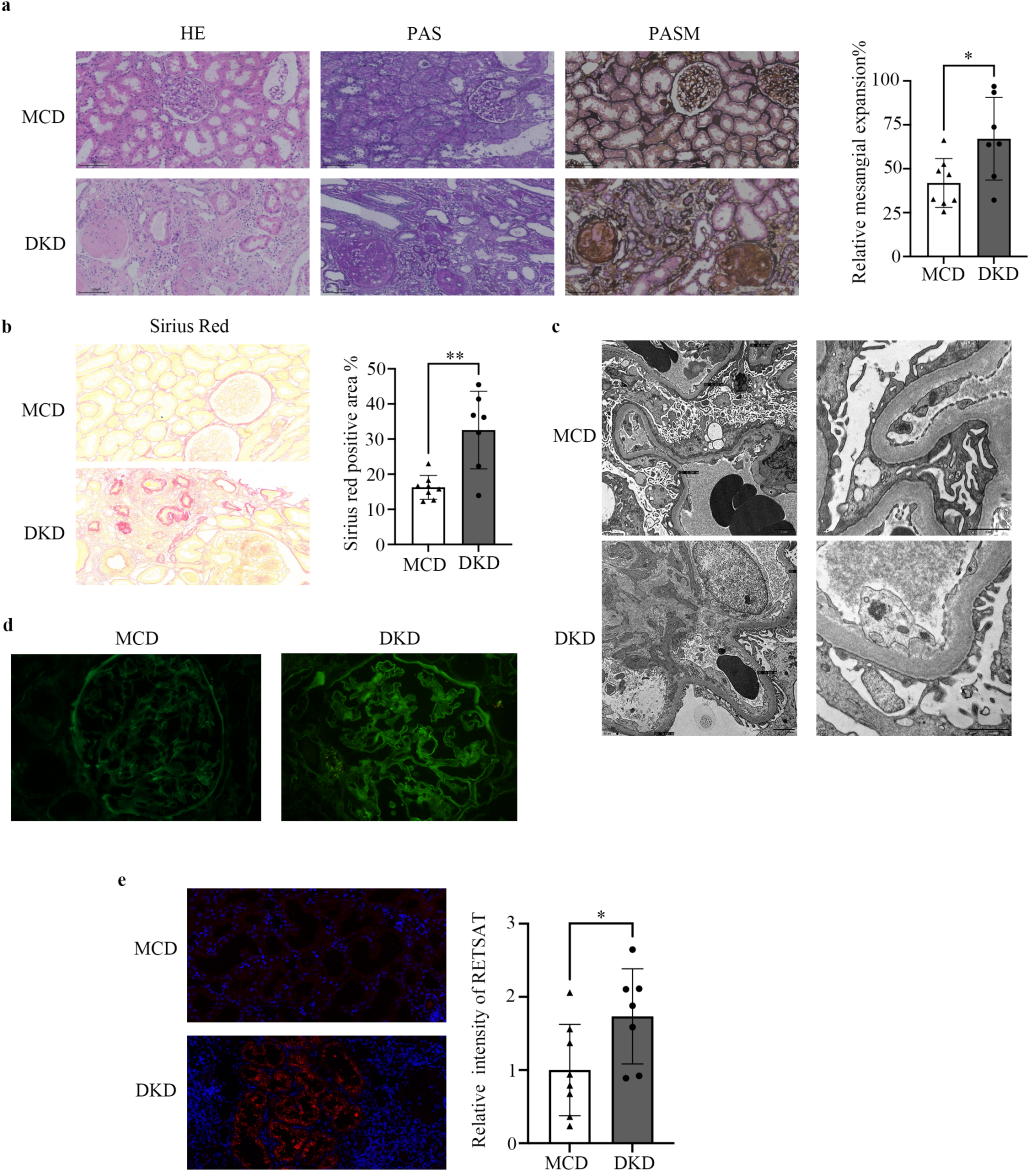
RetSat is highly expressed in renal tubules of DKD patients. **(a)** Representative HE, PAS, and PASM staining of kidney sections from the MCD and DKD groups, alongside relative quantitative analysis of mesangial expansion. Magnification: 200×. **(b)** Sirius Red staining and quantitative analysis of the positive staining area in kidney sections. **(c)** Representative transmission electron microscopy (TEM) images of kidney ultrastructure in the MCD and DKD groups. Magnification: 8,000× and 30,000×. **(d)** Immunofluorescence staining of IgG deposition in kidney sections. Magnification: 400×. **(e)** Immunofluorescence staining of RetSat (red) and nuclei (DAPI, blue) in kidney sections; semi-quantitative analysis of RetSat staining intensity was performed using ImageJ. Data are expressed as mean ± SD. *p < 0.05, **p < 0.01 vs. MCD group.

In contrast, histopathological analysis revealed significant disparities. MCD patients predominantly presented with an interstitial fibrosis and tubular atrophy (IFTA) score of 1, whereas DKD patients exhibited significantly higher scores (42.9% scored 2, 57.1% scored 3; p<0.001). Tubular injury scores also differed significantly: 50.0% of MCD patients showed no injury (score 0), while 57.1% of DKD patients presented with severe injury (score 3; p=0.047). H&E, PAS, and PASM staining demonstrated pronounced mesangial expansion in the DKD group compared to the MCD group, indicative of severe structural injury (**Figure 2a**). Furthermore, Sirius Red staining revealed a significant increase in the fibrotic area in DKD kidneys, confirming the presence of TIF (**Figure 2b**).

TEM revealed distinct ultrastructural features: the MCD group showed extensive foot process effacement and microfilament aggregation, whereas the DKD group exhibited characteristic glomerular basement membrane (GBM) thickening and mild mesangial matrix expansion (**Figure 2c**). Immunofluorescence analysis showed minimal IgG deposition in the MCD group, limited to occasional reabsorption droplets within podocytes. In contrast, the DKD group exhibited positive linear IgG staining along the glomerular capillary walls (**Figure 2d**). Moreover, immunofluorescence staining revealed that RetSat protein expression was significantly elevated in the tubular region of DKD patients compared to the MCD group (**Figure 2e**), suggesting a potential role for RetSat in DKD pathogenesis.

To further explore the cellular landscape and cell-specific *RETSAT* expression, we re-analyzed publicly available snRNA-seq datasets (GSE195460, GSE131882, GSE151302) comprising kidney samples from 6 normal controls (NC) and 5 DKD patients. Clustering analysis of 39,176 cells identified 16 major renal cell types, visualized via uniform manifold approximation and projection (UMAP) (**Supplementary Figure S2A**). Subsequent analysis revealed that *RETSAT* was significantly up-regulated specifically in proximal tubule (PT) cells in the DKD group, exhibiting both a higher proportion of *RETSAT*-positive cells and elevated average expression levels compared to controls (**Supplementary Figure S2B**). Analysis of cellular composition across the 11 samples further detailed the relative abundance of these 16 cell types in the NC and DKD groups (**Supplementary Figure S2C**).

### RetSat is up-regulated in HG-stimulated HK2 cells and can regulate DKD-related TIF

**Figure 3:**
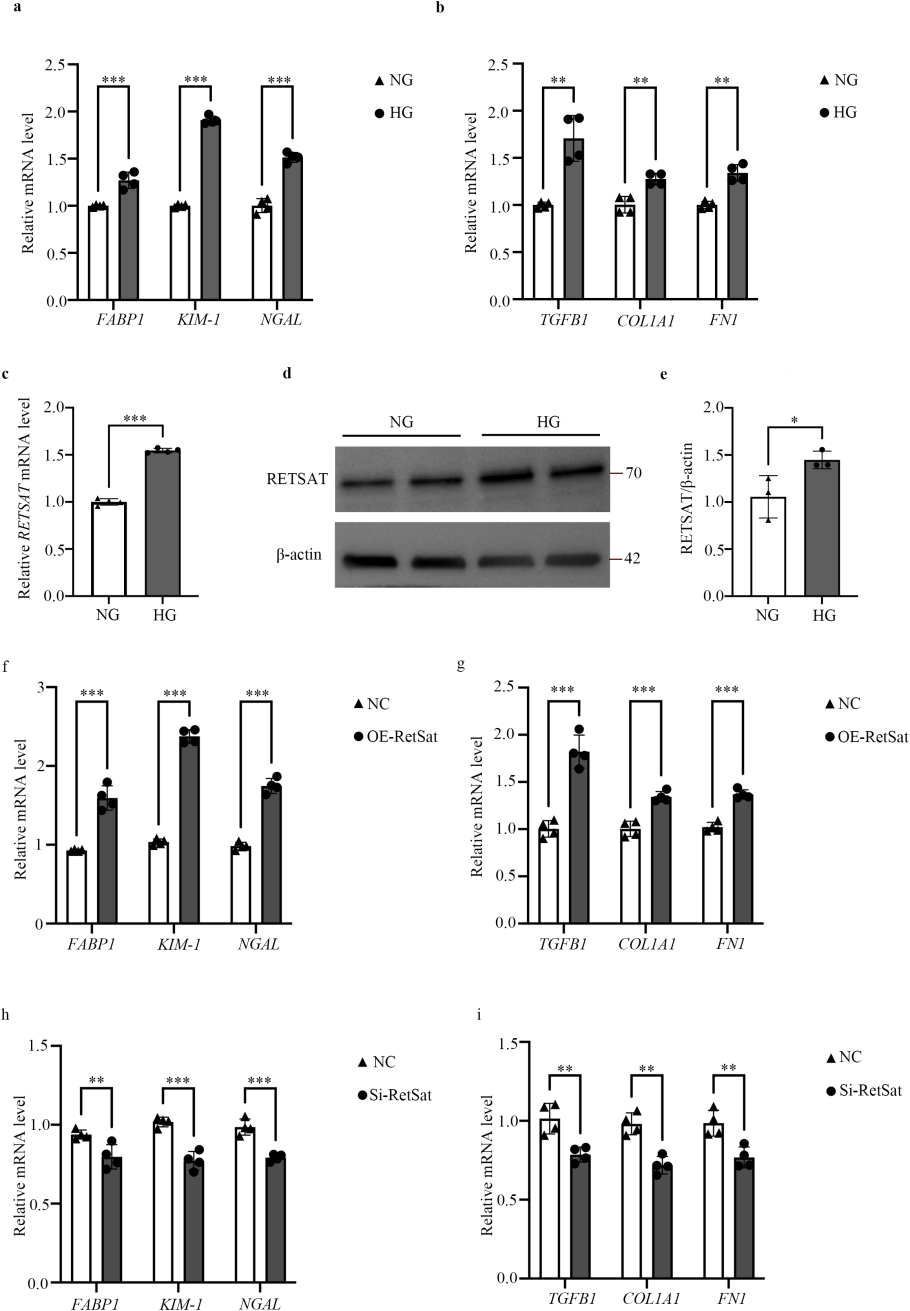
RetSat is up-regulated in HG-stimulated HK2 cells and regulates DKD-related TIF. **(a, b)** Relative mRNA expression of tubular injury markers (*FABP1*, *KIM-1*, *NGAL*) **(a)** and fibrosis markers (*TGFβ1*, *COL1A1*, *FN1*) **(b)** in HK2 cells treated with NG or HG. **(c)** Relative mRNA expression of *RETSAT* in NG- and HG-treated HK2 cells. **(d)** Representative Western blot of RetSat protein expression in NG- and HG-treated HK2 cells. **(e)** Semi-quantitative analysis of RetSat protein levels (n=3). **(f, g)** Relative mRNA expression of tubular injury markers **(f)** and fibrosis markers **(g)** in HG-treated HK2 cells following 48 hours of transfection with Flag-RetSat (OE-RetSat) or empty vector (NC). **(h, i)** Relative mRNA expression of tubular injury markers **(h)** and fibrosis markers **(i)** in HG-treated HK2 cells following 48 hours of transfection with RetSat siRNA (Si-RetSat) or negative control siRNA (NC). Data are presented as mean ± SD. *p < 0.05, **p < 0.01, ***p < 0.001 vs. the respective control group (NG or NC).

To investigate the molecular mechanisms *in vitro*, we established a DKD cell model by stimulating HK2 cells with HG. Validation of this model revealed that the mRNA levels of tubular injury markers (*FABP1*, *KIM-1*, *NGAL*) and fibrosis markers (*COL1A1*, *FN1*, *TGFβ1*) were significantly elevated in HG-stimulated cells compared to the NG control (**Figures 3a, b**). Consistent with our *in vivo* findings, both *RETSAT* mRNA (**Figure 3c**) and RetSat protein levels (**Figures 3d, e**) were significantly increased in HG-stimulated HK2 cells. Based on the up-regulation of RetSat in both DKD renal tubules and HG-stimulated cells, we hypothesized that RetSat drives tubular injury and TIF.

To further verify the regulatory effect of RetSat on TIF, we performed gain- of-function and loss-of-function assays using RetSat overexpression vectors and siRNA, respectively. In HG-stimulated HK2 cells, RetSat overexpression further exacerbated tubular injury and TIF, significantly up-regulating the expression of fibrosis markers (*COL1A1*, *FN1*, *TGFβ1*) and tubular injury markers (*FABP1*, *KIM-1*, *NGAL*) (**Figures 3f, g**). Conversely, RetSat knockdown effectively reversed these trends, significantly down-regulating the expression of the aforementioned markers (**Figures 3h, i**). These findings demonstrate that RetSat positively regulates the progression of DKD-associated TIF *in vitro*.

### Identification of key RetSat targets in DKD pathogenesis

To elucidate the mechanisms underlying RetSat-mediated TIF in DKD, we performed immunoprecipitation-mass spectrometry (IP-MS) in HEK293T cells overexpressing RetSat to identify its binding partners. Among the candidate interactors, we identified Smurf2, a HECT-type E3 ubiquitin ligase with established roles in fibrosis (27). Smurf2 is known to modulate the TGF-β/SMAD signaling cascade, a central driver of extracellular matrix (ECM) accumulation in renal fibrosis (28, 29). Furthermore, Smurf2 has been shown to mitigate fibrosis in DKD by targeting the transcription factor ChREBP for ubiquitination-mediated degradation (30).

Support for this regulation is provided by the PhosphoSitePlus database, which identifies specific ubiquitination sites on ChREBP (**Supplementary Figure S3**). Consequently, we hypothesized that RetSat modulates DKD-associated TIF by interacting with Smurf2 to regulate ChREBP ubiquitination and stability.

### RetSat up-regulates ChREBP in HK2 cells by down-regulating Smurf2

**Figure 4:**
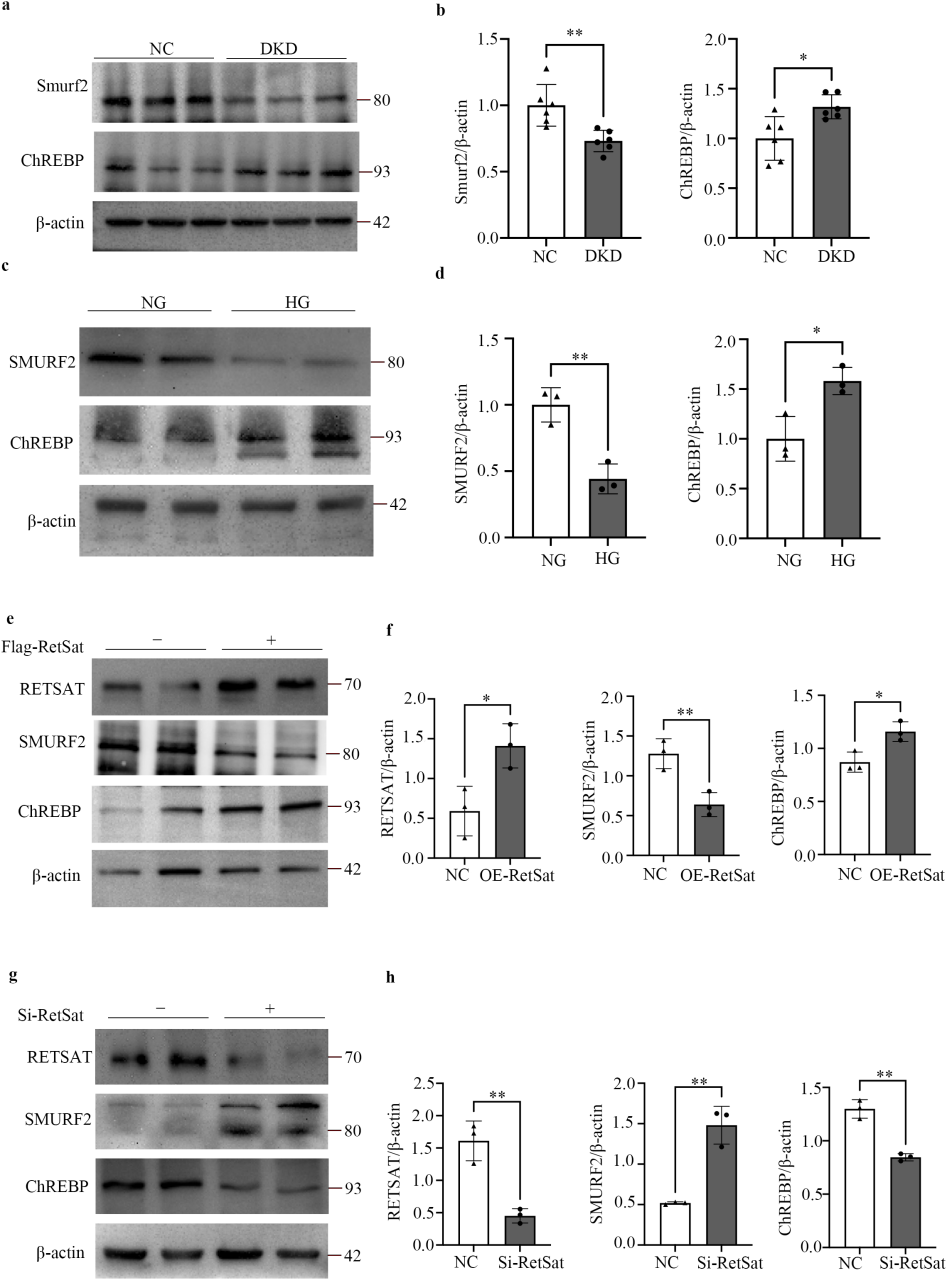
RetSat up-regulates ChREBP and down-regulates Smurf2 expression in DKD mouse kidneys and HG-stimulated HK2 cells. **(a)** Representative Western blots of Smurf2 and ChREBP protein levels in kidney tissues from the NC and DKD groups. **(b)** Semi-quantitative analysis of Smurf2 and ChREBP protein levels in kidney tissues. **(c)** Representative Western blots of Smurf2 and ChREBP protein levels in HK2 cells treated with NG or HG. **(d)** Semi-quantitative analysis of Smurf2 and ChREBP protein levels in HK2 cells. **(e)** Representative Western blots of RetSat, Smurf2, and ChREBP levels in HK2 cells 48 h after transfection with Flag-RetSat (OE-RetSat) or control vector (NC). **(f)** Semi-quantitative analysis of RetSat, Smurf2, and ChREBP protein levels in the OE-RetSat and NC groups. **(g)** Representative Western blots of RetSat, Smurf2, and ChREBP levels in HK2 cells 48 h after transfection with RetSat siRNA (Si-RetSat) or negative control siRNA (NC). **(h)** Semi-quantitative analysis of RetSat, Smurf2, and ChREBP protein levels in the Si-RetSat and NC groups. Data are presented as mean ± SD. *p < 0.05, **p < 0.01 vs. the respective control group (NC or NG).

*In vivo* analysis revealed that Smurf2 protein levels were significantly down-regulated, while ChREBP levels were markedly up-regulated in the kidneys of DKD mice compared to NC mice (**Figures 4a, b**). Consistently, HK2 cells stimulated with HG exhibited reduced Smurf2 and increased ChREBP protein levels compared to the NG group (**Figures 4c, d**). To elucidate the regulatory relationship among RetSat, Smurf2, and ChREBP, we assessed Smurf2 and ChREBP expression in HK2 cells following RetSat modulation. RetSat overexpression significantly suppressed Smurf2 and elevated ChREBP protein levels (**Figures 4e, f**). Conversely, siRNA-mediated knockdown of RetSat resulted in significantly increased Smurf2 levels and decreased ChREBP expression (**Figures 4g, h**). These findings suggest that RetSat functions in renal tubular cells by negatively regulating Smurf2, thereby increasing ChREBP expression.

### RetSat-induced tubular injury and fibrosis are Smurf2-dependent

**Figure 5:**
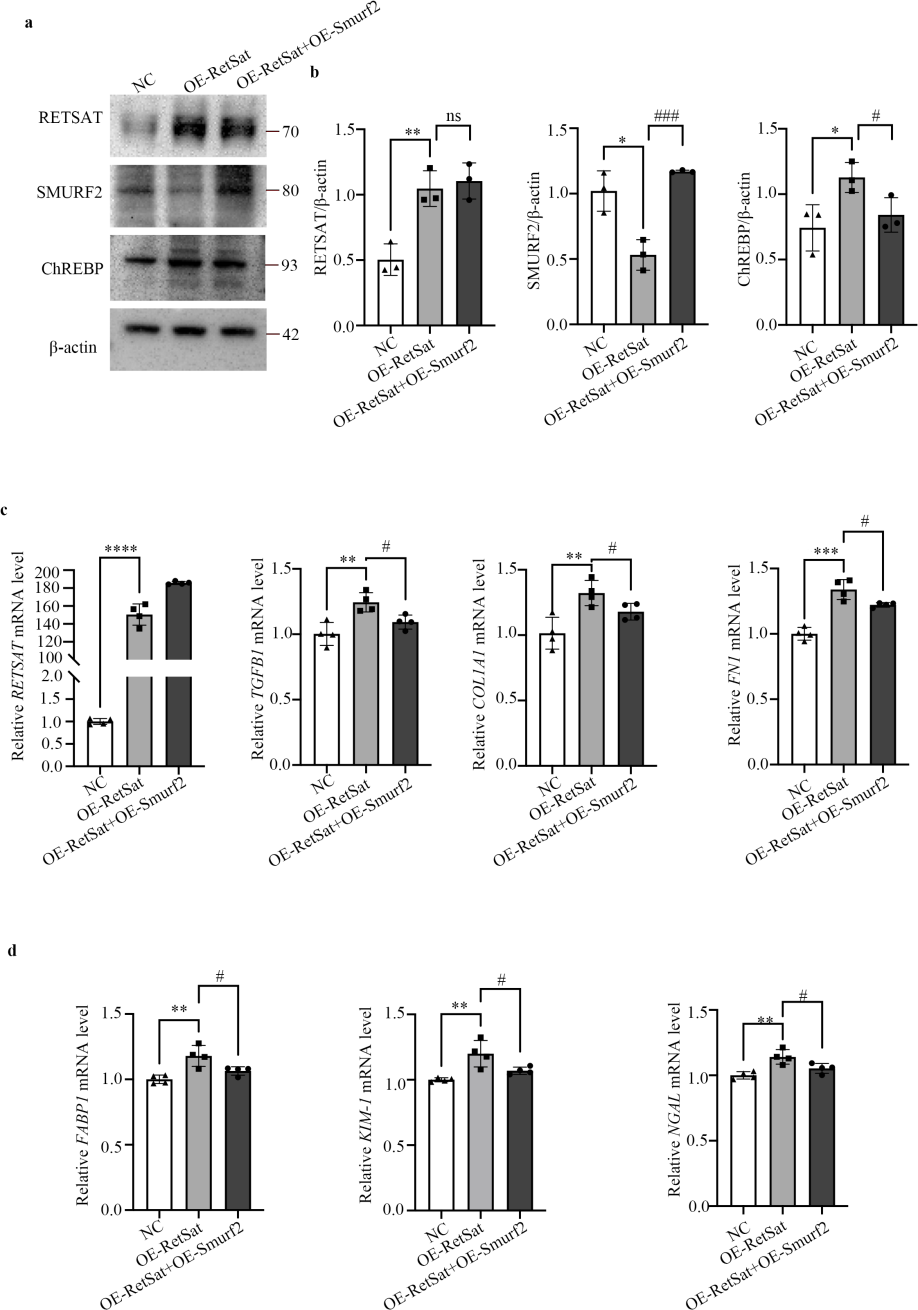
RetSat promotes the expression of ChREBP, fibrosis-associated factors, and renal tubular injury markers by down-regulating Smurf2 in HK2 cells. HK2 cells were transfected with Flag-RetSat vector alone (OE-RetSat) or co-transfected with Flag-RetSat and Ha-Smurf2 vectors (OE-RetSat+OE-Smurf2) for 48 h. **(a)** Representative Western blots of RetSat, Smurf2, and ChREBP protein levels in the NC, OE-RetSat, and OE-RetSat+OE-Smurf2 groups. **(b)** Semi-quantitative analysis of RetSat, Smurf2, and ChREBP protein levels. **(c)** Relative mRNA expression of *RETSAT* and fibrosis markers (*TGFβ1*, *COL1A1*, *FN1*). **(d)** Relative mRNA expression of tubular injury markers (*FABP1*, *KIM-1*, *NGAL*). Data are presented as mean ± SD. *p < 0.05, **p < 0.01, ***p < 0.001, ****p < 0.0001 vs. NC group; #p < 0.05, ###p < 0.001 vs. OE-RetSat group.

To determine whether RetSat-mediated TIF is dependent on Smurf2, we performed rescue experiments by co-transfecting HK2 cells with RetSat and Smurf2 overexpression vectors. As expected, RetSat overexpression alone significantly increased ChREBP protein levels and up-regulated the mRNA expression of fibrosis markers (*COL1A1*, *FN1*, *TGFβ1*) and tubular injury markers (*FABP1*, *KIM-1*, *NGAL*) compared to controls (**Figure 5**). Importantly, co-overexpression of Smurf2 effectively rescued these phenotypes, significantly attenuating both the RetSat-induced increase in ChREBP expression and the associated upregulation of fibrosis and tubular injury markers (**Figure 5**). These results confirm that RetSat drives tubular injury and fibrosis in a Smurf2-dependent manner.

### RetSat reduces Smurf2 protein expression by inducing its ubiquitination and degradation

**Figure 6:**
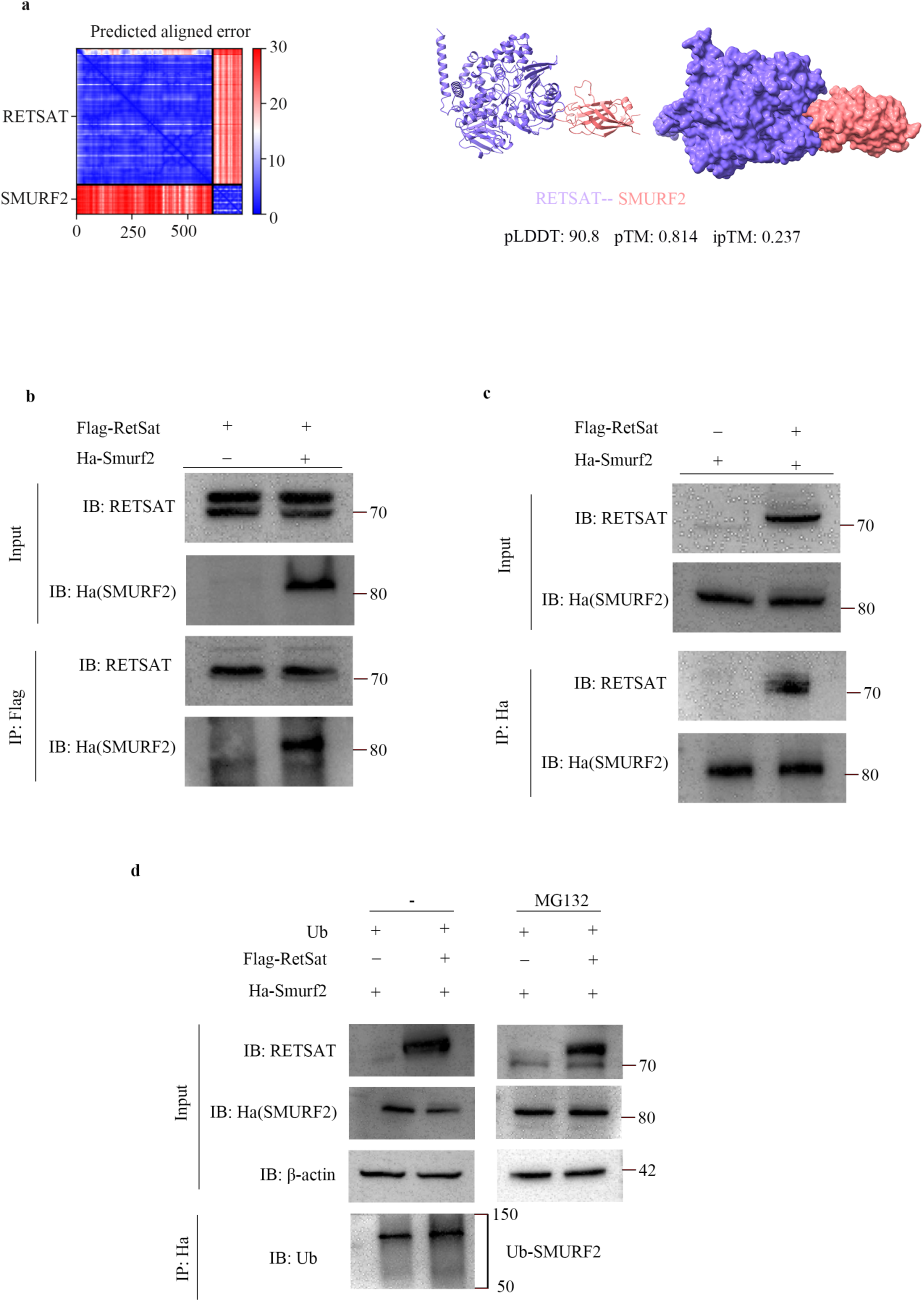
RetSat reduces Smurf2 protein expression by inducing Smurf2 ubiquitination and degradation. **(a)** AlphaFold2 prediction of the interaction between RetSat and Smurf2. **(b, c)** Co-IP analysis of the RetSat-Smurf2 interaction. HEK293T cells were transfected with Flag-RetSat and Ha-Smurf2 vectors for 48 h. Lysates were immunoprecipitated with **(b)** anti-Flag antibody or **(c)** anti-Ha antibody (reciprocal Co-IP), followed by Western blotting with the indicated antibodies. Representative blots from three independent experiments are shown. **(d)** Ubiquitination assay. HEK293T cells were transfected with Ha-Smurf2 and Myc-Ub vectors, in the presence or absence of Flag-RetSat, for 38 h. Cells were subsequently treated with or without MG132 (10 μM) for 10 h. Lysates were immunoprecipitated with anti-Ha antibody, and ubiquitinated Smurf2 (Ub-Smurf2) levels were detected by immunoblotting with anti-Ub antibody. Representative blots from three independent experiments are shown.

To elucidate the regulatory mechanism of RetSat on Smurf2, we first modeled the potential interaction between the two proteins using AlphaFold2 (**Figure 6a**). The predicted structure demonstrated high confidence, with predicted Local Distance Difference Test (pLDDT) scores exceeding 90 and a predicted Template Modeling (pTM) score of 0.814, suggesting a reliable global fold. Although the inter-protein TM (ipTM) score of 0.237 necessitated experimental verification, the low Predicted Aligned Error (PAE) values indicated high structural coherence (31). To biologically validate this interaction, we performed reciprocal Co-IP assays in HEK293T cells. The results confirmed that Flag-RetSat physically interacts with Ha-Smurf2, as demonstrated by their co-precipitation in both experimental directions (**Figures 6b, c**). Given that Smurf2 is subject to ubiquitination (32–34) and possesses documented ubiquitination sites (PhosphoSitePlus, **Supplementary Figure S4**), we investigated whether RetSat promotes Smurf2 degradation via this pathway. HEK293T cells were co-transfected with Ha-Smurf2 and Myc-Ub in the presence or absence of Flag-RetSat. Immunoprecipitation analysis revealed that RetSat overexpression significantly enhanced the polyubiquitination of Smurf2 compared to the control group (**Figure 6d**). Furthermore, treatment with the proteasome inhibitor MG132 effectively blocked RetSat-induced Smurf2 degradation (**Figure 6d**). Collectively, these findings indicate that RetSat promotes Smurf2 degradation via the ubiquitin-proteasome pathway.

### RetSat does not directly regulate ChREBP ubiquitination

**Figure 7:**
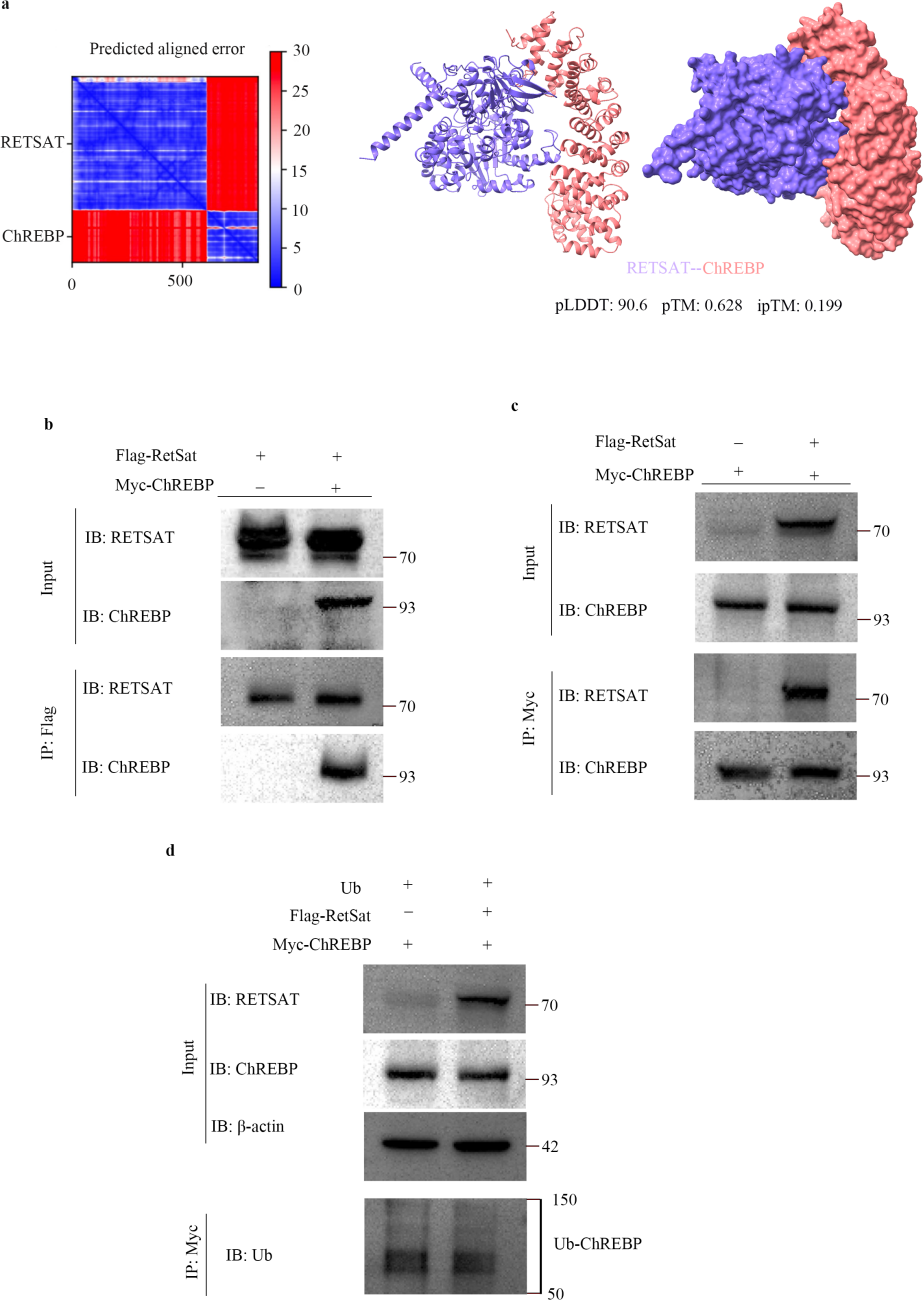
RetSat does not directly regulate ubiquitination of ChREBP. **(a)** AlphaFold2 prediction of the interaction between RetSat and ChREBP. **(b, c)** Co-IP analysis of the RetSat-ChREBP interaction. HEK293T cells were transfected with Flag-RetSat and Myc-ChREBP vectors for 48 h. Lysates were immunoprecipitated with **(b)** anti-Flag antibody or **(c)** anti-Myc antibody (reciprocal Co-IP), followed by Western blotting with the indicated antibodies. Representative blots from three independent experiments are shown. **(d)** Ubiquitination assay. HEK293T cells were co-transfected with Myc-ChREBP and Myc-Ub vectors, in the presence or absence of Flag-RetSat, for 48 h. Lysates were immunoprecipitated with anti-Myc antibody, and ubiquitinated ChREBP (Ub-ChREBP) levels were detected by Western blotting with anti-Ub antibody. Representative blots from three independent experiments are shown.

Although RetSat is known to regulate ChREBP in the pathogenesis of non-alcoholic fatty liver disease (NAFLD), the precise molecular mechanism remains undefined (21). We initially modeled the potential RetSat-ChREBP interaction using AlphaFold2 (**Figure 7a**). The model displayed high confidence (pLDDT > 90) and structural coherence (low PAE), with a pTM score of 0.628 suggesting a plausible fold; however, an ipTM score of 0.199 necessitated experimental verification. Subsequent reciprocal Co-IP assays confirmed a physical interaction between RetSat and ChREBP (**Figures 7b, c**). We next investigated whether this interaction drives ChREBP ubiquitination. In contrast to our findings with Smurf2, RetSat overexpression did not significantly alter ChREBP ubiquitination levels compared to controls (**Figure 7d**). These results indicate that RetSat does not directly catalyze ChREBP ubiquitination, suggesting that its regulatory effect on ChREBP is likely indirect and mediated via Smurf2.

### Smurf2 reduces ChREBP expression by promoting its ubiquitination

**Figure 8:**
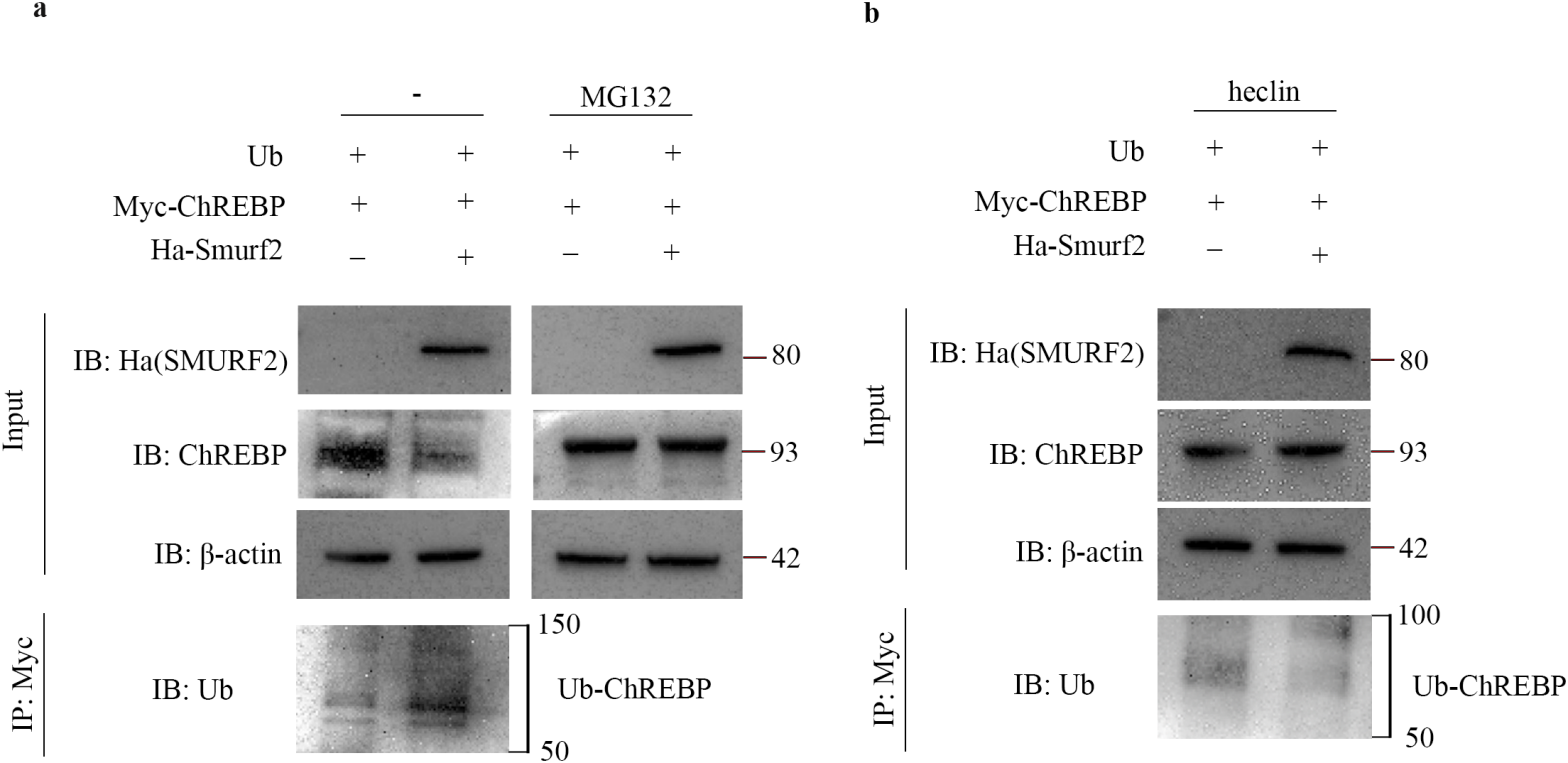
Smurf2 reduces ChREBP expression by promoting its ubiquitination. **(a)** HEK293T cells were co-transfected with Myc-ChREBP and Myc-Ub vectors, in the presence or absence of Ha-Smurf2, for 38 h, followed by treatment with or without MG132 (10 μM) for 10 h. **(b)** HEK293T cells were co-transfected with Myc-ChREBP and Myc-Ub vectors, in the presence or absence of Ha-Smurf2, for 46 h, followed by treatment with heclin (10 μM) for 2 h. **(a, b)** Cell lysates were immunoprecipitated with anti-Myc antibody, and ubiquitinated ChREBP (Ub-ChREBP) levels were detected by Western blotting with anti-Ub antibody. Representative blots from three independent experiments are shown.

To confirm whether RetSat regulates ChREBP indirectly via Smurf2, we first established the direct effect of Smurf2 on ChREBP stability. Overexpression of Smurf2 significantly enhanced ChREBP ubiquitination compared to controls (**Figure 8a**). Furthermore, treatment with the proteasome inhibitor MG132 blocked Smurf2-induced ChREBP degradation (**Figure 8a**), Confirming that Smurf2 promotes ChREBP turnover via the ubiquitin-proteasome pathway. To validate the requirement for Smurf2 ligase activity, we utilized heclin, a specific inhibitor of HECT domain E3 ligases (35). We observed that heclin treatment significantly attenuated Smurf2-mediated ChREBP ubiquitination and degradation (**Figure 8b**). Collectively, these results demonstrate that Smurf2 down-regulates ChREBP protein levels by driving its ubiquitination and subsequent proteasomal degradation.

## Discussion

This study provides the first evidence that RetSat regulates DKD-associated TIF through a ubiquitin-dependent mechanism, offering a novel perspective on its pathogenic role in DKD. We observed that RetSat is significantly up-regulated in the renal tubules of DKD patients and drives both tubular injury and fibrosis. Mechanistically, we identified ChREBP, a key transcriptional regulator of glycolysis, adipogenesis, and renal fibrosis (30, 36, 37), as a downstream effector. Although RetSat interacts with ChREBP, our data indicate that it does not directly catalyze ChREBP ubiquitination but instead indirectly regulates ChREBP stability by modulating the E3 ubiquitin ligase Smurf2. Importantly, we established the causal relationship of this pathway through rigorous loss-of-function and gain-of-function experiments coupled with corresponding rescue assays. Restoring Smurf2 expression effectively reversed RetSat-induced pro-fibrotic phenotypes, confirming that RetSat-driven fibrosis is dependent on Smurf2 suppression. Consequently, we have defined a novel regulatory cascade wherein RetSat suppresses Smurf2 activity, thereby stabilizing ChREBP to promote fibrosis. These findings underscore the critical role of ubiquitination in DKD pathogenesis and highlight this pathway as a potential therapeutic target.

RetSat is an oxidoreductase originally characterized for converting retinol to 13,14-dihydroretinol, with established roles in adipocyte differentiation, hepatic metabolism, macrophage function, and reactive oxygen species (ROS) production (21, 38). In the liver, RetSat depletion reduces triglyceride levels by interfering with cytosolic ChREBP activity independent of retinol conversion, positioning RetSat as a key upstream regulator of hepatic metabolism (21). Furthermore, RetSat is critical for cellular redox homeostasis. Its overexpression increases ROS and lipid peroxidation, whereas its depletion attenuates oxidative stress (19, 39). Recently, RetSat has also been implicated in ferroptosis, promoting iron-dependent cell death via the production of 13,14-dihydroretinol (39). Despite extensive research in liver and adipose tissue, the function of RetSat in renal pathology remains largely unexplored. Our study addresses this gap by confirming significant upregulation of RetSat in renal tubular tissues during DKD progression. Notably, our analysis of human renal biopsies (**Figure 2**) and single-nucleus RNA sequencing data (**Supplementary Figure S2**) consistently revealed significantly elevated RetSat expression in the proximal tubules of DKD patients. Cross-species validation in both human DKD patients and mouse DKD models reinforces the translational medical significance of our findings and supports the clinical value of targeting RetSat to alleviate renal fibrosis.

The pathogenesis of DKD involves complex pathways, including hyperglycemia-induced oxidative stress, inflammation, and activation of the renin-angiotensin-aldosterone system (RAAS) (40). These processes drive extracellular matrix (ECM) components deposition, disrupt renal architecture and promote TIF (41). TIF is a hallmark of DKD, driven by sustained activation of pro-fibrotic signaling cascades. Recent studies continue to uncover novel targets in this landscape: for instance, research published in 2025 highlighted the protective roles of Calycosin (42) and Formononetin (43) in modulating renal fibrosis. Central to fibrotic progression is the TGF-β signaling pathway. As the predominant isoform (44), TGF-β1 transmits signals through transmembrane serine/threonine kinase receptors, phosphorylating Smad proteins. Subsequently, these Smads translocate to the nucleus to drive the expression of fibrotic genes (45).

Emerging evidence suggests that dysregulation of the ubiquitin-proteasome system (UPS), particularly dysfunction of the E3 ubiquitin ligase Smurf2, is pivotal in DKD fibrosis. Smad proteins are the primary substrates of Smurf2, which targets them for ubiquitination and degradation (45). However, the role of Smurf2 in fibrosis is context-dependent. While some studies suggest Smurf2 exacerbates fibrosis by degrading the inhibitory Smad7 (46), others indicate a protective role. Kim et al. demonstrated that Smurf2 attenuates lysophosphatidic acid (LPA)-induced mesangial fibrosis by promoting the ubiquitination and degradation of ChREBP (30). The importance of SMURF2-mediated ChREBP regulation was further emphasized by a recent study showing that miR-1225-3p promotes glomerular fibrosis via the ARHGAP5/SMURF2 pathway (47). Our study identifies RetSat as a novel upstream regulator of this SMURF2-ChREBP axis in renal tubules. Furthermore, while Smurf2 is well-known for inhibiting TGF-β signaling by targeting receptor-regulated Smads (e.g., Smad2/3) for degradation (48), our findings reveal a distinct, TGF-β-independent mechanism. We demonstrate that RetSat promotes the ubiquitination and degradation of Smurf2 itself, which subsequently stabilizes ChREBP to drive TIF. This positions the RetSat-Smurf2-ChREBP axis as a critical, dynamic regulator of DKD fibrosis.

## Limitations and future directions

Although this study provides valuable insights, several limitations warrant consideration. First, ethical constraints precluded kidney biopsies in healthy individuals, resulting in a lack of kidney tissue samples from the general population. Consequently, MCD was used as the control group; however, since MCD is also characterized by massive proteinuria and associated kidney damage, this may limit its value as a control. Second, the human cohort was relatively small and restricted to the Chinese population; future multi-center studies with large-scale, diverse ethnic cohorts are warranted to validate the diagnostic and therapeutic potential of RetSat globally. Third, the models used (STZ-induced HFD mice and human biopsies from DKD stages 1–4) primarily represent early-to-moderate disease stages of DKD, failing to fully replicate ESRD. Whether RetSat exerts similar pro-fibrotic effects in terminal DKD requires further investigation. Fourth, due to the lack of specific small-molecule RetSat inhibitors, this study employed genetic approaches (siRNA) for validation. While this method offers high specificity, developing pharmacological inhibitors would greatly facilitate translational applications. Furthermore, while we focused on the ubiquitination mechanism within the renal tubules, RetSat may also contribute to podocyte injury or endothelial dysfunction through alternative pathways, such as oxidative stress, hypoxia response, or lipid metabolism, which merit exploration in future studies. Finally, *in vivo* validation of the specific regulatory mechanisms identified here remains a priority for subsequent research.

## Conclusion

In conclusion, this study establishes a critical link between RetSat and DKD-associated TIF, identifying for the first time the RetSat-Smurf2-ChREBP pathway as a key driver of DKD progression (**Supplementary Figure S5**). Our findings elucidate the molecular mechanisms by which RetSat regulates renal fibrosis, expanding our understanding of its function beyond metabolic regulation. By highlighting the pivotal role of ubiquitination in this process, this study suggests that targeting the RetSat-Smurf2-ChREBP axis offers a promising therapeutic strategy for the treatment of diabetic kidney disease.

## Data Availability

The original contributions presented in the study are included in the article/**Supplementary Material**. Further inquiries can be directed to the corresponding authors.

## Glossary

TIF: tubulointerstitial fibrosis
DKD: diabetic kidney disease
RetSat: Retinol Saturase
HG: high glucose
FN1: fibronectin
TGF-β1: transforming growth factor beta 1
COL1A1: collagen type 1 alpha 1 chain
HK2: human renal proximal tubular cells
Co-IP: Co-immunoprecipitation
Smurf2: SMAD ubiquitination regulator 2
ChREBP: carbohydrate response element binding protein
CKD: chronic kidney disease
ESRD: end-stage renal disease
ECM: extracellular matrix
PT: proximal tubule
eGFR: estimated glomerular filtration rate
PPARα: peroxisome proliferator-activated receptor α
FOXO1: forkhead boxO1
T2DM: type 2 diabetes mellitus
NC: normal control
HFD: high-fat diet
STZ: streptozotocin
DMEM/F12: Dulbecco’s Modified Eagle Medium/Nutrient Mixture F-12 Ham
FBS: fetal bovine serum
HEK293T: Human embryonic kidney cells
NG: normal glucose
OE: overexpression
PBS: phosphate buffered saline
RIPA: radioimmunoprecipitation assay
Ub: ubiquitin
PDB: Protein Data Bank
MCD: minimal change disease
H&E: Hematoxylin and eosin
PAS: Periodic Acid-Schiff
PASM: Periodic Acid-Silver Methenamine
LFQ: Label-Free Quantification
FASP: filter-aided sample preparation
LC-MS/MS: liquid chromatography-tandem mass spectrometry
HPLC: high-performance liquid chromatography
AGC: automatic gain control
HCD: high-energy collisional dissociation
FABP1: fatty acid binding protein 1
KIM-1: kidney injury molecule-1
NGAL: neutrophil gelatinase-associated lipocalin
DEPs: differentially expressed proteins
BMI: body mass index
HbA1c: hemoglobin A1c
UACR: urine albumin-to-creatinine ratio
IFTA: interstitial fibrosis and tubular atrophy
IP-MS: immunoprecipitation-mass spectrometry
PLDDT: predicted local distance difference test
pTM: predicted template modeling
ipTM: inter-protein TM
PTM: post-translational modification
EMT: epithelial-to-mesenchymal transition
UPS: ubiquitin-proteasome system
LPA: lysophosphatidic acid
NAFLD: non-alcoholic fatty liver disease
IB: immunoblotting
MG132: N-carbobenzoxy-L-leucinyl-L-leucinal
BUN: blood urea nitrogen
Scr: serum creatinine
EDNO: endothelial cell
PT: epithelial cell of proximal tubule
FIB: fibroblast
DCT: kidney distal convoluted tubule epithelial cell
TAL: kidney loop of Henle thick ascending limb epithelial cell
ALT: kidney loop of Henle thin ascending limb epithelial cell
LEUK: leukocyte
MES: mesangial cell
PEC: parietal epithelial cell
PODO: podocyte
ICA: renal alpha-intercalated cell
ICB: renal beta-intercalated cell
PC: renal principal cell

## References

[1] Tuttle KR Bakris GL Bilous RW Chiang JL de Boer IH Goldstein-Fuchs J . Diabetic kidney disease: a report from an ADA Consensus Conference. Diabetes Care. (2014) 37:2864–83. doi: 10.2337/dc14-1296, PMID: 25249672 PMC4170131

[2] Chen X Chen C Tian X He L Zuo E Liu P . DBAN: An improved dual branch attention network combined with serum Raman spectroscopy for diagnosis of diabetic kidney disease. Talanta. (2024) 266:125052. doi: 10.1016/j.talanta.2023.125052, PMID: 37574605

[3] GBD 2021 Diabetes Collaborators . Global, regional, and national burden of diabetes from 1990 to 2021, with projections of prevalence to 2050: a systematic analysis for the Global Burden of Disease Study 2021. Lancet. (2023) 402:203–34. doi: 10.1016/S0140-6736(23)01301-6, PMID: 37356446 PMC10364581

[4] Thomas MC Brownlee M Susztak K Sharma K Jandeleit-Dahm KAM Zoungas S . Diabetic kidney disease. Nat Rev Dis Primers. (2015) 1:15018. doi: 10.1038/nrdp.2015.18, PMID: 27188921 PMC7724636

[5] Humphreys BD . Mechanisms of renal fibrosis. Annu Rev Physiol. (2018) 80:309–26. doi: 10.1146/annurev-physiol-022516-034227, PMID: 29068765

[6] Li L Fu H Liu Y . The fibrogenic niche in kidney fibrosis: components and mechanisms. Nat Rev Nephrol. (2022) 18:545–57. doi: 10.1038/s41581-022-00590-z, PMID: 35788561

[7] Gilbert RE Cooper ME . The tubulointerstitium in progressive diabetic kidney disease: more than an aftermath of glomerular injury? Kidney Int. (1999) 56:1627–37. doi: 10.1046/j.1523-1755.1999.00721.x, PMID: 10571771

[8] Xu C Ha X Yang S Tian X Jiang H . Advances in understanding and treating diabetic kidney disease: focus on tubulointerstitial inflammation mechanisms. Front Endocrinol (Lausanne). (2023) 14:1232790. doi: 10.3389/fendo.2023.1232790, PMID: 37859992 PMC10583558

[9] Chevalier RL . The proximal tubule is the primary target of injury and progression of kidney disease: role of the glomerulotubular junction. Am J Physiol Renal Physiol. (2016) 311:F145–61. doi: 10.1152/ajprenal.00164.2016, PMID: 27194714 PMC4967168

[10] Popovic D Vucic D Dikic I . Ubiquitination in disease pathogenesis and treatment. Nat Med. (2014) 20:1242–53. doi: 10.1038/nm.3739, PMID: 25375928

[11] Akhouri V Majumder S Gaikwad AB . The emerging insight into E3 ligases as the potential therapeutic target for diabetic kidney disease. Life Sci. (2023) 321:121643. doi: 10.1016/j.lfs.2023.121643, PMID: 36997061

[12] Wu W Huang XR You Y Xue L Wang X-J Meng X . Latent TGF-β1 protects against diabetic kidney disease via Arkadia/Smad7 signaling. Int J Biol Sci. (2021) 17:3583–94. doi: 10.7150/ijbs.61647, PMID: 34512167 PMC8416717

[13] Chen Y-C Wu M-Y Yu Z-L Chou W-H Lai Y-T Kao C-C . Association of UBE3C variants with reduced kidney function in patients with diabetic kidney disease. J Pers Med. (2020) 10:210. doi: 10.3390/jpm10040210, PMID: 33171965 PMC7712123

[14] Moise AR Kuksa V Imanishi Y Palczewski K . Identification of all-trans-retinol:all-trans-13,14-dihydroretinol saturase. J Biol Chem. (2004) 279:50230–42. doi: 10.1074/jbc.M409130200, PMID: 15358783 PMC2665716

[15] Schupp M Lefterova MI Janke J Leitner K Cristancho AG Mullican SE . Retinol saturase promotes adipogenesis and is downregulated in obesity. Proc Natl Acad Sci U.S.A. (2009) 106:1105–10. doi: 10.1073/pnas.0812065106, PMID: 19139408 PMC2633572

[16] Sun Y Ng L Lam W Lo CK-C Chan P-T Yuen Y-L . Identification and characterization of a novel mouse peroxisome proliferator-activated receptor alpha-regulated and starvation-induced gene, Ppsig. Int J Biochem Cell Biol. (2008) 40:1775–91. doi: 10.1016/j.biocel.2008.01.006, PMID: 18289917

[17] Shin D-J Joshi P Hong S-H Mosure K Shin D-G Osborne TF . Genome-wide analysis of FoxO1 binding in hepatic chromatin: potential involvement of FoxO1 in linking retinoid signaling to hepatic gluconeogenesis. Nucleic Acids Res. (2012) 40:11499–509. doi: 10.1093/nar/gks932, PMID: 23066095 PMC3526316

[18] Park PJ Kong SW Tebaldi T Lai WR Kasif S Kohane IS . Integration of heterogeneous expression data sets extends the role of the retinol pathway in diabetes and insulin resistance. Bioinformatics. (2009) 25:3121–7. doi: 10.1093/bioinformatics/btp559, PMID: 19786482 PMC2778339

[19] Pang X-Y Wang S Jurczak MJ Shulman GI Moise AR . Retinol saturase modulates lipid metabolism and the production of reactive oxygen species. Arch Biochem Biophys. (2017) 633:93–102. doi: 10.1016/j.abb.2017.09.009, PMID: 28927883 PMC5659944

[20] Sarang Z Joós G Garabuczi É Rühl R Gregory CD Szondy Z . Macrophages engulfing apoptotic cells produce nonclassical retinoids to enhance their phagocytic capacity. J Immunol. (2014) 192:5730–8. doi: 10.4049/jimmunol.1400284, PMID: 24850721

[21] Heidenreich S Witte N Weber P Goehring I Tolkachov A von Loeffelholz C . Retinol saturase coordinates liver metabolism by regulating ChREBP activity. Nat Commun. (2017) 8:384. doi: 10.1038/s41467-017-00430-w, PMID: 28855500 PMC5577314

[22] Mirdita M Schütze K Moriwaki Y Heo L Ovchinnikov S Steinegger M . ColabFold: making protein folding accessible to all. Nat Methods. (2022) 19:679–82. doi: 10.1038/s41592-022-01488-1, PMID: 35637307 PMC9184281

[23] Pettersen EF Goddard TD Huang CC Meng EC Couch GS Croll TI . UCSF ChimeraX: Structure visualization for researchers, educators, and developers. Protein Sci. (2021) 30:70–82. doi: 10.1002/pro.3943, PMID: 32881101 PMC7737788

[24] Zhang B Ye W Ye Y Zhou H Saeed A Chen J . Structural insights into Cas13b-guided CRISPR RNA maturation and recognition. Cell Res. (2018) 28:1198–201. doi: 10.1038/s41422-018-0109-4, PMID: 30425321 PMC6274644

[25] Ma Z Li L Livingston MJ Zhang D Mi Q Zhang M . p53/microRNA-214/ULK1 axis impairs renal tubular autophagy in diabetic kidney disease. J Clin Invest. (2020) 130:5011–26. doi: 10.1172/JCI135536, PMID: 32804155 PMC7456229

[26] Wilson PC Muto Y Wu H Karihaloo A Waikar SS Humphreys BD . Multimodal single cell sequencing implicates chromatin accessibility and genetic background in diabetic kidney disease progression. Nat Commun. (2022) 13:5253. doi: 10.1038/s41467-022-32972-z, PMID: 36068241 PMC9448792

[27] Rotin D Kumar S . Physiological functions of the HECT family of ubiquitin ligases. Nat Rev Mol Cell Biol. (2009) 10:398–409. doi: 10.1038/nrm2690, PMID: 19436320

[28] Wang L Zha H Huang J Shi L . Flavin containing monooxygenase 2 regulates renal tubular cell fibrosis and paracrine secretion via SMURF2 in AKI−CKD transformation. Int J Mol Med. (2023) 52:110. doi: 10.3892/ijmm.2023.5313, PMID: 37800598 PMC10558214

[29] Zou J Zhou X Ma Y Yu R . Losartan ameliorates renal interstitial fibrosis through metabolic pathway and Smurfs-TGF-β/Smad. BioMed Pharmacother. (2022) 149:112931. doi: 10.1016/j.biopha.2022.112931, PMID: 36068784

[30] Kim D Nam G-Y Seo E Jun H-S . Inhibition of ChREBP ubiquitination via the ROS/Akt-dependent downregulation of Smurf2 contributes to lysophosphatidic acid-induced fibrosis in renal mesangial cells. J BioMed Sci. (2022) 29:31. doi: 10.1186/s12929-022-00814-1, PMID: 35538534 PMC9092836

[31] Elfmann C Stülke J . PAE viewer: a webserver for the interactive visualization of the predicted aligned error for multimer structure predictions and crosslinks. Nucleic Acids Res. (2023) 51:W404–10. doi: 10.1093/nar/gkad350, PMID: 37140053 PMC10320053

[32] Ogunjimi AA Briant DJ Pece-Barbara N Le Roy C Di Guglielmo GM Kavsak P . Regulation of Smurf2 ubiquitin ligase activity by anchoring the E2 to the HECT domain. Mol Cell. (2005) 19:297–308. doi: 10.1016/j.molcel.2005.06.028, PMID: 16061177

[33] Zhang W Dai J Hou G Liu H Zheng S Wang X . SMURF2 predisposes cancer cell toward ferroptosis in GPX4-independent manners by promoting GSTP1 degradation. Mol Cell. (2023) 83:4352–69. doi: 10.1007/s00018-022-04601-x, PMID: 38016474

[34] Yuan B Liu J Cao J Yu Y Zhang H Wang F . PTPN3 acts as a tumor suppressor and boosts TGF-β signaling independent of its phosphatase activity. EMBO J. (2019) 38:e99945. doi: 10.15252/embj.201899945, PMID: 31304624 PMC6627230

[35] Mund T Lewis MJ Maslen S Pelham HR . Peptide and small molecule inhibitors of HECT-type ubiquitin ligases. Proc Natl Acad Sci U.S.A. (2014) 111:16736–41. doi: 10.1073/pnas.1412152111, PMID: 25385595 PMC4250122

[36] Xu X Mendoza A Krumm CS Su S Acuña M Bare CJ . ChREBP-mediated up-regulation of Them1 coordinates thermogenesis with glycolysis and lipogenesis in response to chronic stress. Sci Signal. (2024) 17:eadk7971. doi: 10.1126/scisignal.adk7971, PMID: 39626011 PMC11817722

[37] Tang Y Wallace M Sanchez-Gurmaches J Hsiao W-Y Li H Lee PL . Adipose tissue mTORC2 regulates ChREBP-driven *de novo* lipogenesis and hepatic glucose metabolism. Nat Commun. (2016) 7:11365. doi: 10.1038/ncomms11365, PMID: 27098609 PMC4844681

[38] Weber P Flores RE Kiefer MF Schupp M . Retinol saturase: more than the name suggests. Trends Pharmacol Sci. (2020) 41:418–27. doi: 10.1016/j.tips.2020.03.007, PMID: 32345479

[39] Bi G Liang J Shan G Bian Y Chen Z Huang Y . Retinol saturase mediates retinoid metabolism to impair a ferroptosis defense system in cancer cells. Cancer Res. (2023) 83:2387–404. doi: 10.1158/0008-5472.CAN-22-3977, PMID: 37184371

[40] Sinha SK Nicholas SB . Pathomechanisms of diabetic kidney disease. J Clin Med. (2023) 12:7349. doi: 10.3390/jcm12237349, PMID: 38068400 PMC10707303

[41] Dugbartey GJ . Diabetic nephropathy: A potential savior with ‘rotten-egg’ smell. Pharmacol Rep. (2017) 69:331–9. doi: 10.1016/j.pharep.2016.11.004, PMID: 28183033

[42] Dalal D Singh L Singh A . Calycosin and kidney health: a molecular perspective on its protective mechanisms. Pharmacol Rep. (2025) 77:658–69. doi: 10.1007/s43440-025-00728-3, PMID: 40249500

[43] Singh A Singh L Dalal D . Formononetin as a multifaceted modulator of renal pathology: insights into fibrotic, oxidative, inflammatory, and apoptotic pathways. Pharmacol Rep. (2025) 78:194–208. doi: 10.1007/s43440-025-00801-x, PMID: 41148562

[44] Chia Z-J Cao Y-N Little PJ Kamato D . Transforming growth factor-β receptors: versatile mechanisms of ligand activation. Acta Pharmacol Sin. (2024) 45:1337–48. doi: 10.1038/s41401-024-01235-6, PMID: 38351317 PMC11192764

[45] Zhang Y Chang C Gehling DJ Hemmati-Brivanlou A Derynck R . Regulation of Smad degradation and activity by Smurf2, an E3 ubiquitin ligase. Proc Natl Acad Sci U.S.A. (2001) 98:974–9. doi: 10.1073/pnas.98.3.974, PMID: 11158580 PMC14694

[46] Yang F Huang XR Chung ACK Hou C-C Lai KN Lan HY . Essential role for Smad3 in angiotensin II-induced tubular epithelial-mesenchymal transition. J Pathol. (2010) 221:390–401. doi: 10.1002/path.2721, PMID: 20593491

[47] Zhang J Cai Y Qin Y Liu J Ding J Xu M . miR-1225-3p regulates fibrosis in mesangial cells via SMURF2-mediated ubiquitination of ChREBP in diabetic kidney disease. Ren Fail. (2025) 47:2484632. doi: 10.1080/0886022X.2025.2484632, PMID: 40211762 PMC11995769

[48] Liu Z Wang W Li X Tang S Meng D Xia W . Capsaicin ameliorates renal fibrosis by inhibiting TGF-β1-Smad2/3 signaling. Phytomedicine. (2022) 100:154067. doi: 10.1016/j.phymed.2022.154067, PMID: 35349832

