# Supplementary material for: Retinol saturase promotes tubulointerstitial fibrosis in diabetic kidney disease by inhibiting ChREBP ubiquitination via Smurf2 suppression
Source: Front Endocrinol (Lausanne). 2026 Feb 4;17:1759785. doi: 10.3389/fendo.2026.1759785 (PMC12913137; doi:10.3389/fendo.2026.1759785)
Supplement: Supplementary file 2 [file DataSheet2.docx]

Table S1. qPCR primer sequences used in the present study.

A, Primer sequences used for mice kidney tissues

Gene Primer sequences (5’ 3’)

Retsat F: GTCTACGTGGGCCTTTACG

R: ACTTTCTTCCTAGCCTCCTTGT

Col1α1 F: GCTCCTCTTAGGGGCCACT

R: CCACGTCTCACCATTGGGG

Fn1 F: AGGAAGCCGAGGTTTTAACTG

R: AGGACGCTCATAAGTGTCACC

Tgfβ1 F: CTCCCGTGGCTTCTAGTGC

R: GCCTTAGTTTGGACAGGATCTG

Kim-1 F: ATCGTGGAATCACAACGACA

R: TGCCCCTTTAAGTTGTACCG

Ngal F: TGGAAGAACCAAGGAGCTGT

R: GGTGGGGACAGAGAAGATGA

Fabp1 F: ATGAACTTCTCCGGCAAGTACC

R: CTGACACCCCCTTGATGTCC

β-actin F: GGCTGTATTCCCCTCCATCG

R CCAGTTGGTAACAATGCCATGT

B, Primer sequences used for human HK2 cells

Gene Primer sequences (5’ 3’)

RetSat F: CAGCATTGGCCGTTTTATCTTG

R: CCTTGAGGCCCTGAATGTAGG

Col1α1 F: GAGGGCCAAGACGAAGACATC

R: CAGATCACGTCATCGCACAAC

Fn1 F: CGGTGGCTGTCAGTCAAAG

R: AAACCTCGGCTTCCTCCATAA

Tgfβ1 F: GGCCAGATCCTGTCCAAGC

R: GTGGGTTTCCACCATTAGCAC

Kim-1 F: TGTCTGGACCAATGGAACCC

R: GGCAACAATATACGCCACTGT

Ngal F: GACAACCAATTCCAGGGGAAG

R: GCATACATCTTTTGCGGGTCT

Fabp1 F: AAGACAGTGGTTCAGTTGGAAG

R: TGAGTTCGGTCACAGACTTGAT

β-actin F: GTGTTGCCCCTGAAGAGCAT

R: GCTGGGACATTGAAAGTCTCA

Table S2. Clinical Characteristics of patients with MCD and patients with DKD

|  | Total (n=15) | MCD (n=8) | DKD (n=7) | p |
| --- | --- | --- | --- | --- |
| Age (year) | 56.0 ± 19.6 | 51.9±23.8 | 60.7±13.5 | 0.402 |
| Sex |  |  |  | 0.569 |
| Male | 11 (73.33) | 5 (62.50) | 6 (85.71) |  |
| Female | 4 (26.67) | 3 (37.50) | 1 (14.29) |  |
| BMI (kg/m^2^) | 25.1 ± 5.2 | 24.3±6.4 | 26.1±3.5 | 0.523 |
| Smoke (n, %) | 4 (26.67) | 2 (25.00) | 2 (28.57) | 1.000 |
| Alcohol use (n, %) | 1 (6.67) | 1 (12.50) | 0 (0.00) | 1.000 |
| Hypertension (n, %) | 10 (66.67) | 4 (50.00) | 6 (85.71) | 0.282 |
| HbA1c (%) | 6.5 ± 1.0 | 6.0±0.4 | 7.1±1.2 | 0.026 |
| Creatinine (μmol/L) | 142.0 (100.3, 161.4) | 119.0 (98.7, 151.4) | 152.7 (116.8, 331.9) | 0.203 |
| Urea nitrogen | 10.50 (6.0, 16.4) | 9.9 (6.2, 16.2) | 16.2 (6.7, 23.0) | 0.694 |
| eGFR (mL/min/1.73m^2^) | 46.2 (17.1, 83.6) | 46.6 (16.4, 100.1) | 35.1 (9.5, 93.6) | 0.779 |
| UACR (mg/g) | 304.3 (71.9, 503.2) | 415.1 (54.8, 620.9) | 206.2 (61.3, 453.7) | 0.536 |
| IFTA score (n, %) |  |  |  | <0.001 |
| 1 | 8 (53.3) | 8 (100.0) | 0 (0.0) |  |
| 2 | 3 (20.0) | 0 (0.0) | 3 (42.9) |  |
| 3 | 4 (26.7) | 0 (0.0) | 4 (57.1) |  |
| Tubular injury score (n, %) |  |  |  | 0.047 |
| 0 | 4 (26.67) | 4 (50.00) | 0 (0.00) |  |
| 1 | 3 (20.00) | 2 (25.00) | 1 (14.29) |  |
| 2 | 4 (26.67) | 2 (25.00) | 2 (28.57) |  |
| 3 | 4 (26.67) | 0 (0.00) | 4 (57.14) |  |

Abbreviations: IFTA: interstitial fibrosis and tubular atrophy


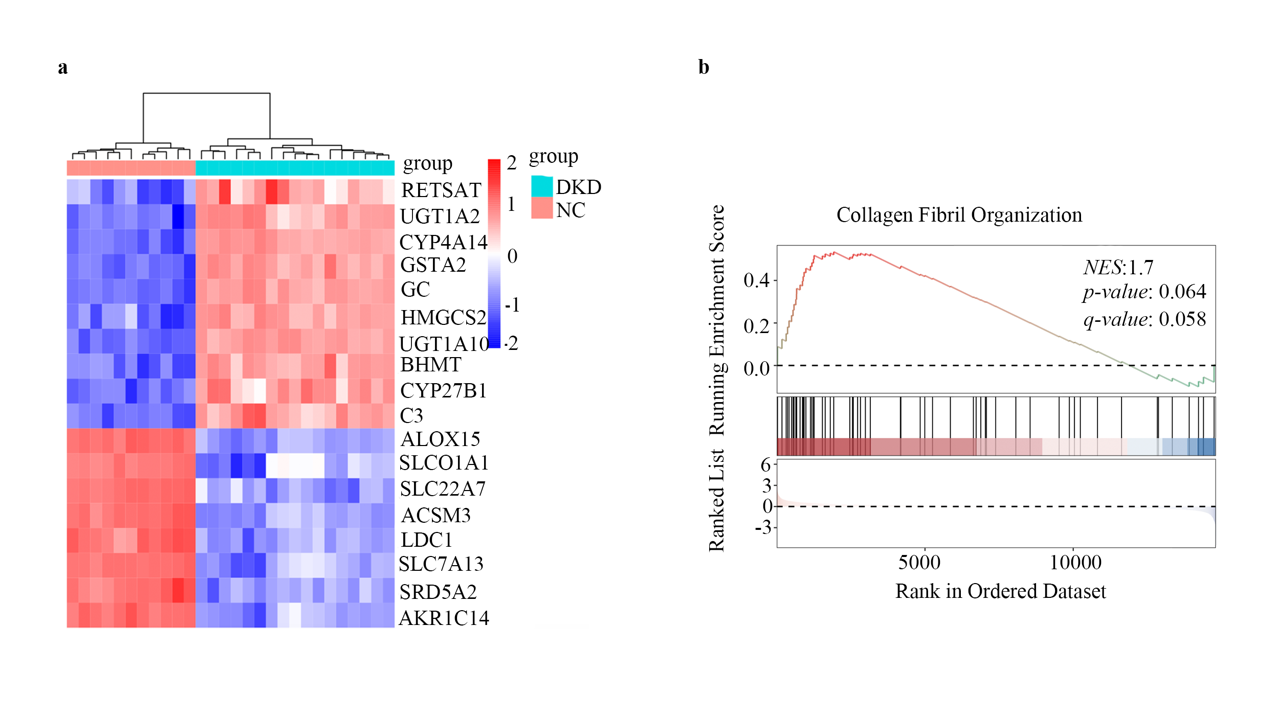


Fig. S1. RetSat expression and fibrosis pathway enrichment in DKD kidneys.

(a) Heatmap comparison of differentially expressed genes from the GSE228960 dataset between NC and DKD groups. (b) GSEA of GSE228960 dataset.


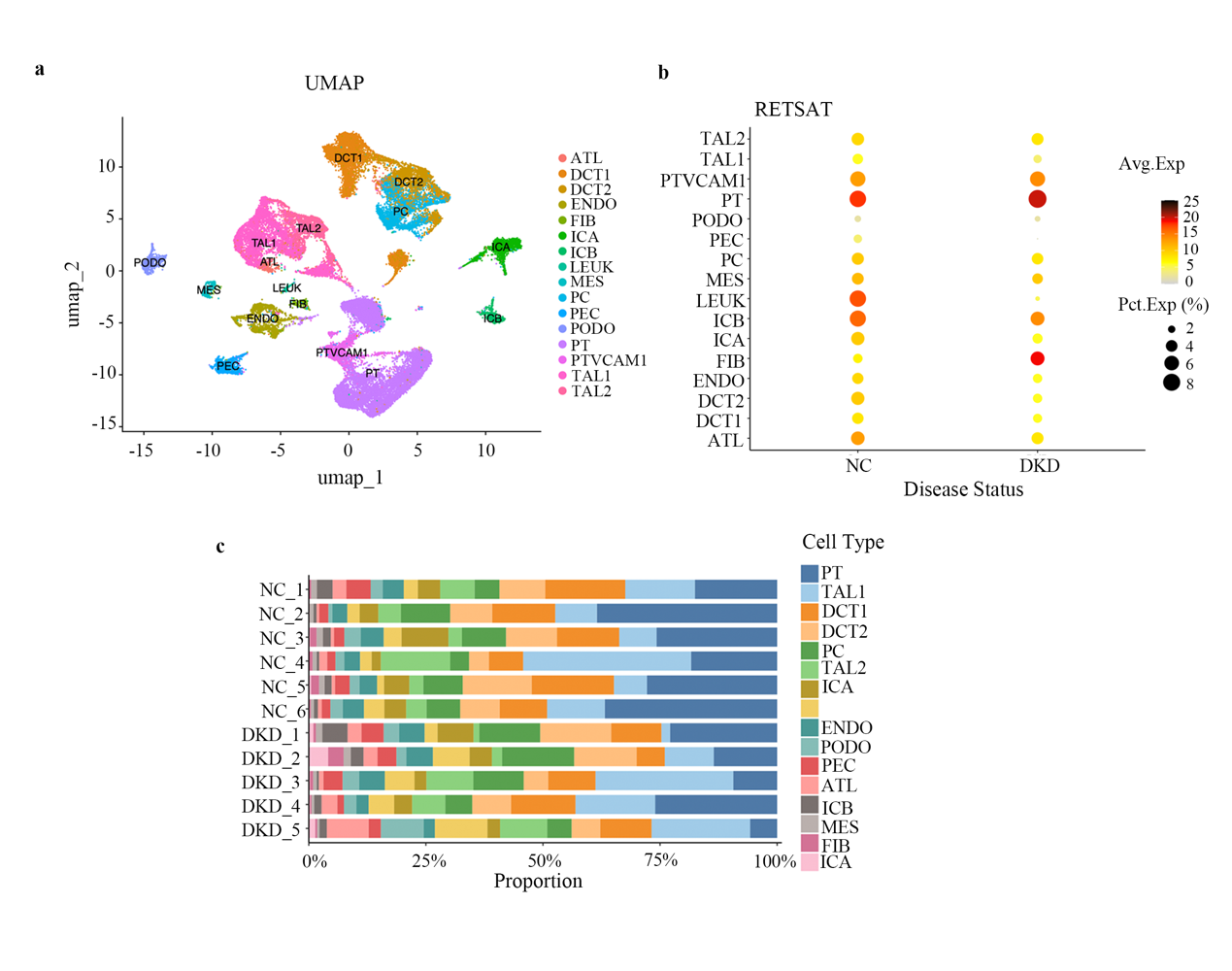


Fig. S2. Cellular landscape and RETSAT expression patterns in human DKD kidneys. (a) Bar plot illustrating the proportion of identified renal cell types for each individual NC (n=6) and DKD (n=5) sample.(b) Dot plot showing the percentage of expressing cells and the average expression level of RETSAT across different cell types in NC vs DKD. (c) UMAP visualization of the single-cell transcriptomic landscape, showing 39,176 high-quality cells aggregated from six NC and five DKD samples. Cells are colored by their annotated cell type, illustrating the major renal cell populations. Abbreviations: UMAP, Uniform Manifold Approximation and Projection; PT, Proximal Tubule; PTVCAM1, Vascular Cell Adhesion Molecule-1 (VCAM1) + PT; PEC, Parietal Epithelial Cell; ATL, Ascending Thin Limb; TAL, Thick Ascending Limb; DCT, Distal Convoluted Tubule; PC, Principal Cell; ICA/B, Intercalated Cell A/B; PODO, Podocyte; ENDO, Endothelial Cell; MES, Mesangial Cell; FIB, Fibroblast; LEUK, Leukocyte.


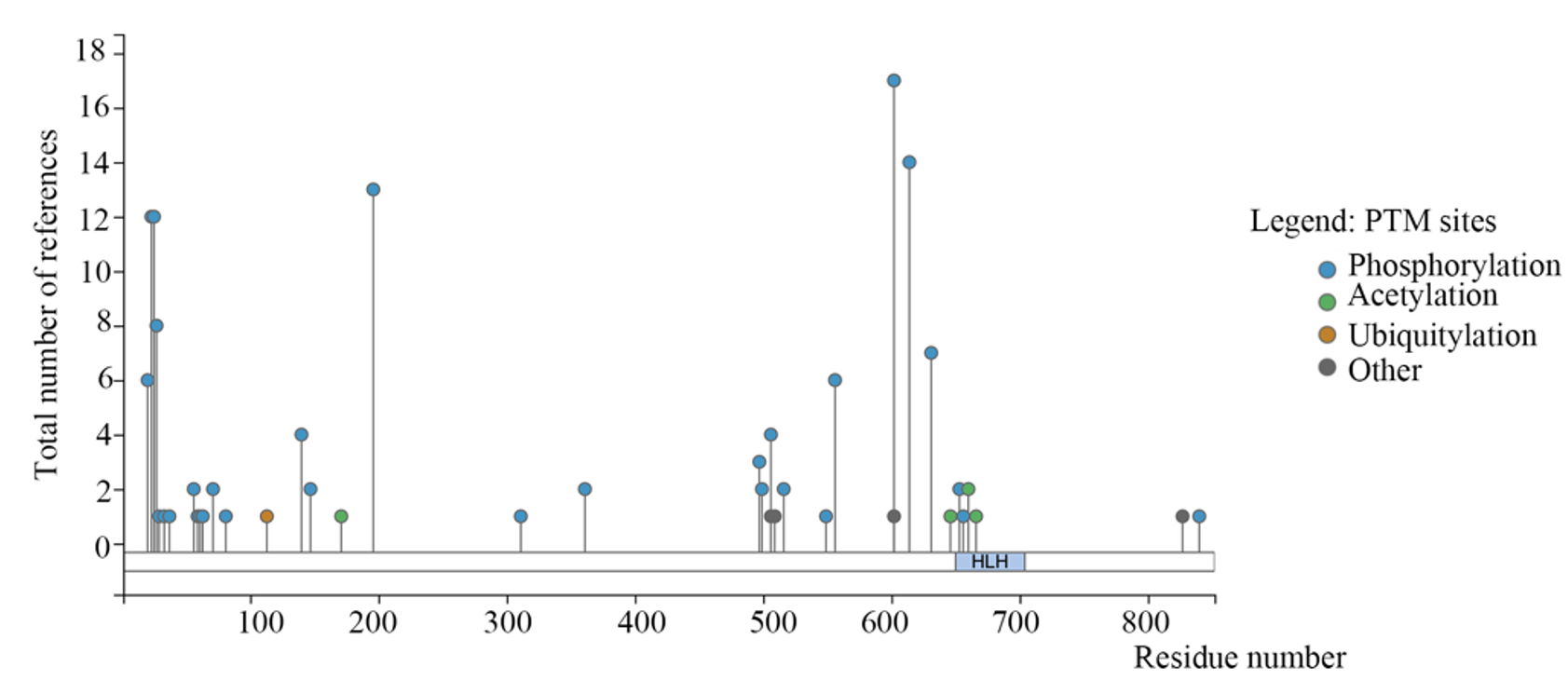

Fig. S3. PTM sites of ChREBP identified using PhosphoSitePlus.


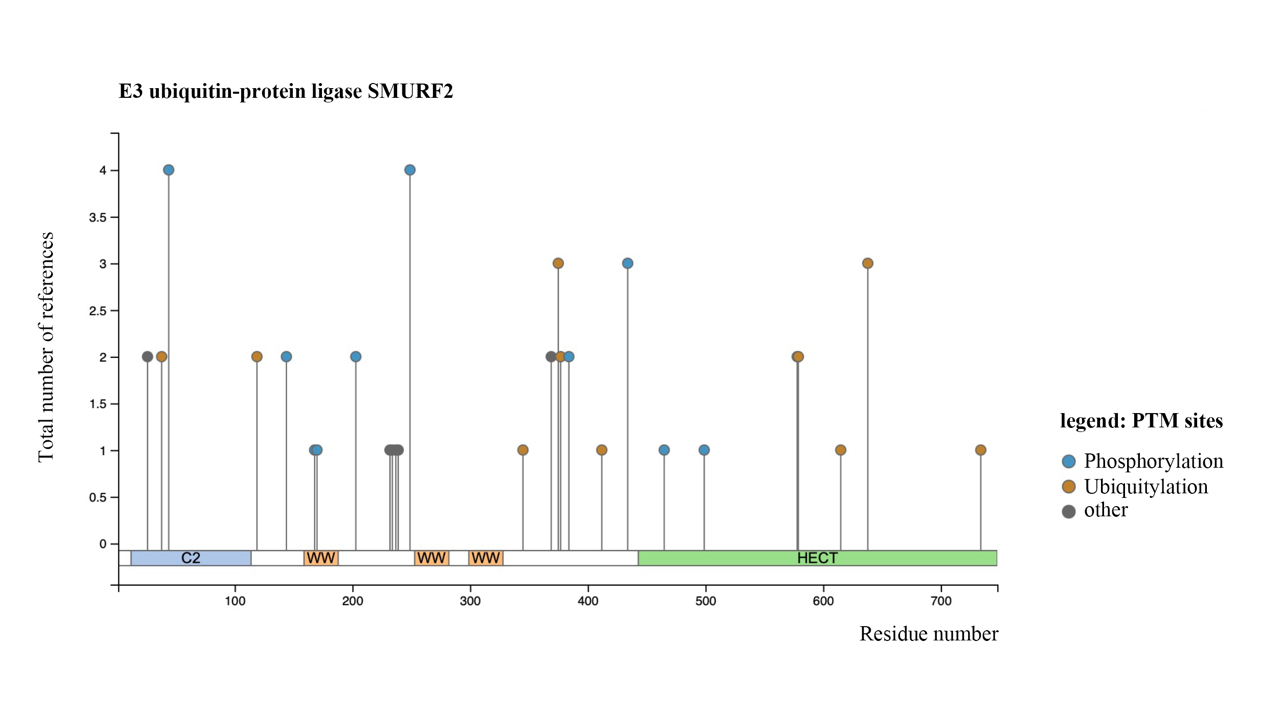


Fig. S4. PTM sites of SMURF2 identified using PhosphoSitePlus.


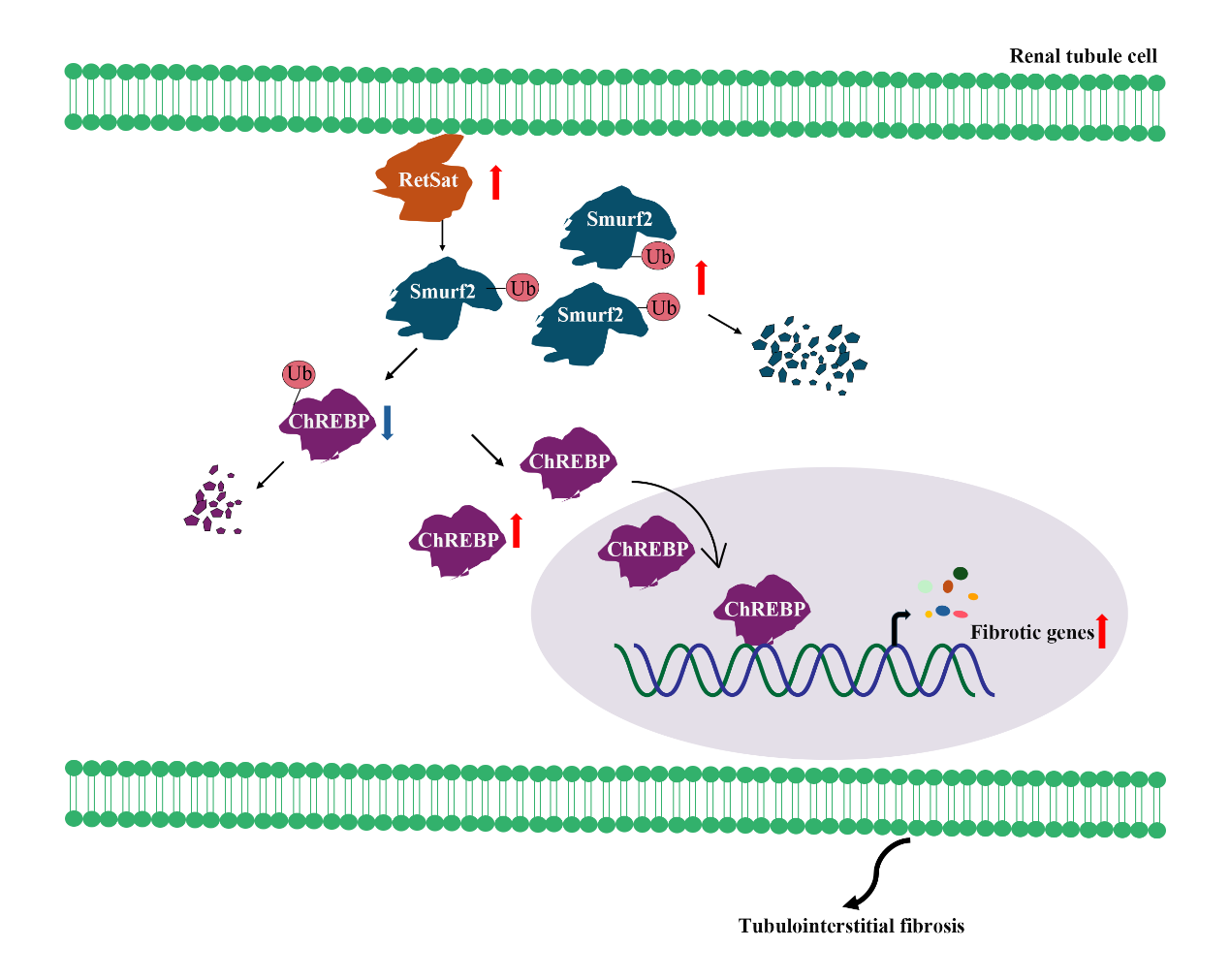


Fig. S5. Schematic representation of the mechanism by which RetSat induces ChREBP-mediated tubulointerstitial fibrosis by downregulating Smurf2 in renal tubular cells. Red and blue arrows indicate up-regulated and down-regulated responses, respectively.

Methods

Human samples

This study was approved by the Ethics Committee of Tongji Hospital, School of Medicine, Tongji University, (Approval No. K-W-2024-009). A total of 15 patients who were diagnosed or treated in the Department of Nephrology of Tongji Hospital participated in this study. Written informed consent was obtained from all participants prior to their participation in the study. Human renal tissue samples obtained by renal puncture were collected from two groups of patients: 1) 7 patients diagnosed with DKD, and 2) 8 patients with minimal change disease (MCD) as controls. DKD and MCD were diagnosed after sample evaluation under light microscopy, direct immunofluorescence and transmission electron microscopy.

Animal study

Eight-week-old male C57BL/6 mice were randomly divided into two groups: the normal control (NC) group (n=6) and the DKD group (n=6). Mice in the NC group were fed a normal chow diet containing 10% kcal fat (Trophic, China), whereas those in the DKD group were fed a high-fat diet (HFD) containing 60% kcal fat (Trophic, China). After 4 weeks of continuous feeding, mice in the DKD group were administered streptozotocin (STZ; 90 mg/kg; Sigma Aldrich, USA) intraperitoneally dissolved in 0.1 mol/L citrate buffer (pH 4.5, Solarbio, China), and an equal volume of citrate buffer was injected into mice in the NC group intraperitoneally. Mice with fasting blood glucose levels exceeding 11.1 mmol/L were considered diabetic. To establish the DKD model, T2DM mice were maintained on a HFD for an additional 12 weeks to induce DKD. After establishing the DKD model, kidneys from both groups of mice were harvested for further analysis. All animal experiments were approved by the Ethics Committee of Tongji Hospital, School of Medicine, Tongji University, and were performed in compliance with the Guidelines for the Care and Use of Experimental Animals at Tongji Hospital. Mice were housed in pathogen-free facilities at Tongji Hospital, with individually ventilated cages, free access to water and food, with a maximum of 5 mice per cage.

Cell culture and treatment

Human renal proximal tubular cells (HK2) were purchased from FuHeng Biotechnology Co., Ltd. (Shanghai, China). Cells were cultured in Dulbecco’s Modified Eagle Medium / Nutrient Mixture F-12 Ham (DMEM/F12 1:1 mixture) with 10% fetal bovine serum (FBS; FH100-900, China). Human embryonic kidney cells (HEK293T) were obtained from the Cell Bank of the Chinese Academy of Sciences (Shanghai, China) and cultured in DMEM (G4511, Servicebio, Wuhan, China) containing 10% FBS. HK2 cells were treated with high glucose (HG, 30 mM) medium to establish an in vitro model of DKD. HK2 cells were seeded in 6-well plates at approximately 70% confluence, starved for 24 hours, and then treated with normal glucose (NG, 5.5 mM) or HG (30 mM) for 72 hours. To investigate whether RetSat regulates ChREBP expression through Smurf2 and the role of Smurf2 in this pathway, HK2 cells were divided into the following groups: 1) Normal control group (NC, empty cDNA vector); 2) overexpression-RetSat (OE-RetSat) group (Flag-RetSat vector + empty cDNA vector); 3) OE-RetSat + OE-Smurf2 group (Flag-RetSat vector + Ha-Smurf2 vector). HK2 cells were transfected with Flag-RetSat vector, Ha-Smurf2 vector, and their negative controls for 48 hours. Overexpression vectors (Flag-RetSat and Ha-Smurf2) were obtained from Hanheng Biotech (Shanghai, China). All vectors were transfected into HK2 cells using Lipo8000™ transfection reagent (C0533, Beyotime, Shanghai, China) according to the manufacturer’s instructions.

siRNA transfection

To investigate the role of RetSat in HK2 cells, gene expression was knocked down using small interfering RNA (siRNA) obtained from Sangon Biotech (Shanghai, China).The siRNA targeting RetSat (5′-CCAACUACUAUGUUUACUATT-3′, 5′-UAGUAAACAUAGUAGUUGGTT-3′) was transfected in HK2 cells using Lipo8000™ transfection reagent (C0533, Beyotime, Shanghai, China) following the manufacturer’s protocol.

Co-immunoprecipitation (Co-IP)

To investigate protein interactions, HEK293T cells were transfected with the indicated vectors for 48 hours. Cells were washed with Phosphate Buffered Saline (PBS) and lysed with RIPA Lysis Buffer (P0013D, Beyotime, Shanghai, China) containing protease inhibitor (G2008, Servicebio, Wuhan, China) for 30 minutes on ice. The lysates were centrifuged at 12,000 rpm for 15 minutes at 4 °C to remove debris. The supernatants were incubated with anti-HA beads (P2121, Beyotime), anti-Myc beads (P2118, Beyotime, Shanghai, China), or anti-Flag beads (P2115, Beyotime, Shanghai, China) overnight at 4 °C for immunoprecipitation. Beads were washed three times with Tris-buffered saline (TBS, G0001, Servicebio, Wuhan, China). The precipitates were boiled in 1× SDS loading buffer at 100 °C for 10 minutes and then analyzed by Western blotting.

Ubiquitination assay

HEK293T cells were co-transfected with Myc-Ubiquitin (Ub), Flag-RetSat, and Ha-Smurf2, and treated with or without 10 μM MG132 for 10 hours before harvest. In another experiment, HEK293T cells were co-transfected with Myc-Ub, Ha-Smurf2, and Myc-ChREBP. These cells were divided into three groups: one group was treated with 10 μM MG132 for 10 hours, one group was treated with 10 μM Heclin for 2 hours, and the other group was an untreated control group. In the third experiment, HEK293T cells were co-transfected with Myc-Ub, Flag-RetSat, and Myc-ChREBP. All cells were treated for a total of 48 hours. Cells were washed with PBS and lysed with RIPA lysis buffer containing protease inhibitors. A total of 1000 μg of cell lysate was incubated at 4 °C overnight with 10 μL anti-Ha beads or anti-Myc beads to pull down Ha- or Myc-tagged proteins, respectively. Beads were washed three times with TBS, and then the proteins were detected by SDS-PAGE. Myc-ChREBP overexpression vector was purchased from Hanheng Biotech (Shanghai, China), and Myc-Ub vector was obtained from OBiO (Shanghai, China). All vectors were transfected using Lipo8000™ transfection reagent.

Western blotting analysis

Proteins were obtained from the kidney or HK2 cells using RIPA lysis buffer (P0013B, Beyotime, Shanghai, China) containing protease inhibitors. Protein concentrations were quantified by BCA Protein Quantification Kit (EC001, SparkJade, Shandong, China). Equal amounts of proteins were separated by sodium dodecyl sulfate-polyacrylamide gel electrophoresis (SDS-PAGE) and transferred to PVDF membrane (IPVH00010, Merck Millipore, Billerica, MA, USA). Membranes were blocked with skimmed milk in TBST for 1 hour at room temperature and then incubated overnight at 4 °C with primary antibodies . Primary antibodies used included anti-RetSat (#PA5-65443, 1:1000, Thermo Fisher Scientific, MA, USA), anti-Smurf2 (#12024S, 1:1000, Cell Signaling Technology, MA, USA), and anti-ChREBP (#13256-1-AP, 1:1000, Proteintech, Wuhan, China). Additionally, anti-Myc (#AF0033, 1:1000, Beyotime, Shanghai, China), anti-Ha (#M20003, 1:1000, Abmart, Shanghai, China), and anti-β-actin (#60008-1-Ig, 1:1000, Proteintech, Wuhan, China) were used. Subsequently, HRP-labeled secondary antibodies, including goat anti-rabbit IgG (#A0208, 1:1000, Beyotime, Shanghai, China) and goat anti-mouse IgG (#A0216, 1:1000, Beyotime, Shanghai, China), were incubated at room temperature for 1 hour.

Quantitative real-time PCR (qPCR)

Total RNA was isolated from kidney tissues or HK2 cells using TRIzol reagent (Invitrogen, CA, USA). cDNA was synthesized from 1000 ng of total RNA using a cDNA reverse transcription Kit (Takara RR037A, Japan). Quantitative PCR (qPCR) amplifications were performed on a QuantStudio7Flex System (ABI, USA) using ChamQ SYBR qPCR Master Mix (Q331-02, Vazyme, Nanjing, China). Gene expression levels were quantified using the 2-∆∆Ct method, with β-actin as an internal control. Specific primers for qPCR amplification were designed and synthesized by Sangon Biotech (Shanghai, China), and their sequences were listed in Supplementary Table S1.

AlphaFold2 prediction

The sequence of RetSat (Q6NUM9) was obtained from UniProtKB, and the sequences of ChREBP and Smurf2 were obtained from the Protein Data Bank (PDB) (6MJL and 2JQZ, respectively). The complete structures of RetSat, ChREBP, and Smurf2 proteins were predicted using AlphaFold on Google Colab with default settings[1, 2]. We compared the top five ranked outputs from AlphaFold based on confidence scores and chose the highest confidence (rank 1) for the graphs.. Structural visualization diagrams were generated using UCSF ChimeraX[3], where protein structures rendered in ribbon or surface representation.

Histological analysis

Kidney tissues from mice and human patients were paraffin-embedded, sectioned, and analyzed histologically. Hematoxylin and eosin (H&E), Periodic Acid-Schiff (PAS), and Periodic Acid-Silver Methenamine (PASM) staining were used to assess morphological changes. To evaluate glomerular cross-sectional area, mesangial expansion, and tubular damage, PASM and PAS-stained sections were analyzed. Six mice per group were randomly selected, and 20 glomeruli per mouse were analyzed for mesangial expansion. Human kidney tissue samples were analyzed in a similar manner. Additionally, 20 randomized regions at the cortical-medullary junction were evaluated for tubular injury using a semi-quantitative scoring system: 0 (normal), 1 (<25% injury), 2 (26–50% injury), and 3 (>50% injury). Mesangial area and tubular injury percentages were quantified using ImageJ software. Sirius Red staining was performed to evaluate renal fibrosis where collagen deposition area was analyzed semi-quantitatively using ImageJ.

Immunofluorescence

Immunofluorescence staining technique was performed as previously described[4]. Kidney sections were stained with RetSat antibody (#PA5-65443, 1:1000, Thermo Fisher Scientific, MA, USA) overnight at 4°C, followed by incubation with Alexa-594 goat anti-rabbit (#111-585-003, Jackson ImmunoResearch, USA). Nuclei were counterstained with DAPI (Boster, USA), and fluorescent signals were visualized using inverted fluorescence microscope (Nikon, Japan).

Biochemical measurements

Following a fasting period of at least 12 hours, blood was collected from mice. The levels of serum creatinine (Scr) and blood urea nitrogen (BUN) were determined using an automated biochemical analyzer (PUZS-300X, Perlong, China) at the Central Clinical Laboratory of Tongji Hospital. Urinary concentrations of kidney injury molecule-1 (KIM-1) were quantified using a commercial ELISA kit (CUSABIO, USA) in accordance with the manufacturer’s protocol.

Label-freequantification (LFQ)-based proteomics analysis

Kidney tissues from three mice in each group were lysed to extract proteins, followed by quantification using the BCA protein assay kit according to the manufacturer’s instructions. Approximately 200 μg of protein per sample was trypsinized using the filter-aided sample preparation (FASP) method, followed by peptide desalting. For liquid chromatography-tandem mass spectrometry (LC-MS/MS) analysis, peptides were desalted on a C18 cartridge, concentrated by vacuum centrifugation, re-dissolved in 40 μL of 0.1% formic acid, and quantified by OD280. The digested peptides were then analyzed by LC-MS/MS as previously reported. Peptides were separated using high-performance liquid chromatography (HPLC) on an Easy-nLC system (Proxeon Biosystems). Peptides were loaded onto a loading column (Thermo Scientific Acclaim PepMap100, 100 μm × 2 cm, nanoViper C18) and then separated on an analytical column (Thermo Scientific EASY column, 10 cm, ID 75 μm, 3 μm, C18-A2) at a flow rate of 300 nL/min. Elution was performed with Buffer B (0.1% formic acid in 84% acetonitrile) over different gradients. The eluted peptides were analyzed using a Q-Exactive mass spectrometer (Thermo Fisher Scientific, USA) with the following parameters: MS1 resolution set to 70,000 at 200 m/z, automatic gain control (AGC) target at 1e6, maximum injection time of 50 ms, and dynamic exclusion duration of 60 s. Each MS2 scan was triggered after a full MS1 scan, 20 fragments per scan were collected using high-energy collisional dissociation (HCD) with an isolation window of 2 m/z, and a MS2 resolution of 17,500 at 200 m/z, a normalized collision energy of 30 eV, and an underfill ratio of 0.1%.

GEO dataset analysis

Gene expression profiles for DKD and control kidney samples were retrieved from the Gene Expression Omnibus (GSE228960). Kidney samples were obtained from uninephrectomized db/db mice and db/m mice. Raw data were imported into R (v4.2.1) and processed using the limma package (v3.50.3) for background correction, quantile normalization, and log₂ transformation of expression values. Gene Set Enrichment Analysis (GSEA) was performed using the clusterProfiler package (v4.2.0) against the Gene Ontology Biological Process gene set (c5.go.bp.v7.4.symbols.gmt) and curated gene sets (c2.all.v2024.1.Hs.symbols.gmt) obtained from the Molecular Signatures Database (MSigDB, v7.4). Pathways with a normalized enrichment score (NES) >1.5, Benjamini–Hochberg–adjusted p‑value <0.05 and a false discovery rate (FDR) q‑value <0.05 were considered significantly enriched.

snRNA-seq data processing and analysis

In this study, we performed a re-analysis of a publicly available human kidney snRNA-seq dataset obtained from the Gene Expression Omnibus (GSE195460, GSE131882, GSE151302)[5]. The dataset comprises six NC and five DKD samples. The analysis was initiated using the pre-processed count matrix and associated metadata file from the original study. A Seurat object was constructed in R (v4.4.3) using the Seurat package (v5.3.0), containing a total of 39,176 single-nuclei profiles after the original authors' quality control and doublet removal. The data was normalized using the NormalizeData function (LogNormalize method, scale factor = 10,000), and the top 2,000 highly variable features were identified using FindVariableFeatures. The expression of all genes was scaled using ScaleData, followed by principal component analysis (PCA) with the RunPCA function. UMAP was subsequently performed on the first 24 principal components to visualize the cellular landscape. For visualization of RETSAT expression, a dot plot was generated using the DotPlot function to show both the percentage of expressing cells and the average expression level. To analyze cellular composition, the proportion of each cell type within each individual sample was calculated using the dplyr package (v1.1.4) and visualized as a stacked bar plot using ggplot2. All data processing and visualization were conducted in the R statistical environment.

Immunoprecipitation mass spectrometry (IP/MS) analysis

HEK293T cells were transfected with either Flag vector or Flag-RetSat plasmid. Anti-Flag beads were added to cell lysates for immunoprecipitation. LC-MS/MS analysis was performed on a Q-Exactive HF mass spectrometer. Mass spectrometry data were analyzed using MaxQuant software version 2.4.14.0 and searched against the UniProt-Reference Proteome-Homo sapiens database. To visualize the overlap between the identified proteins and the TGF-β signaling pathway genes, a Venn diagram was constructed. Specifically, proteins interacting with RetSat were compared to the set of TGF-β pathway-related genes (KEGG_TGF_BETA_SIGNALING_PATHWAY.v2024.1.Hs.gmt) to identify shared and distinct elements.

Statistical analysis

Statistical analyses were conducted using SPSS software (Version 27.0; IBM, USA), R software (Version 4.2.1, The R Foundation; http://www.R-project.org), and GraphPad Prism 9.0 (GraphPad Software, Inc., La Jolla, CA, USA). Data were expressed as means ± standard deviations (SD). Differences between two groups were evaluated using the unpaired, two-tailed Student's t-test, assuming a normal distribution of the data. p-values < 0.05 were considered statistically significant, and all tests were two-sided.

References

1. Zhang B, Ye W, Ye Y, Zhou H, Saeed AFUH, Chen J, et al. Structural insights into Cas13b-guided CRISPR RNA maturation and recognition. Cell Res. 2018;28(12):1198-201.

2. Mirdita M, Schütze K, Moriwaki Y, Heo L, Ovchinnikov S, Steinegger M. ColabFold: making protein folding accessible to all. Nat Methods. 2022;19(6):679-82.

3. Pettersen EF, Goddard TD, Huang CC, Meng EC, Couch GS, Croll TI, et al. UCSF ChimeraX: Structure visualization for researchers, educators, and developers. Protein Sci. 2021;30(1):70-82.

4. Ma Z, Li L, Livingston MJ, Zhang D, Mi Q, Zhang M, et al. p53/microRNA-214/ULK1 axis impairs renal tubular autophagy in diabetic kidney disease. J Clin Invest. 2020;130(9):5011-26.

5. Wilson PC, Muto Y, Wu H, Karihaloo A, Waikar SS, Humphreys BD. Multimodal single cell sequencing implicates chromatin accessibility and genetic background in diabetic kidney disease progression. Nat Commun. 2022 Sep 6;13(1):5253.
